# Long-read genomics reveal extensive nuclear-specific evolution and allele-specific expression in a dikaryotic fungus

**DOI:** 10.1101/gr.280359.124

**Published:** 2025-06

**Authors:** Rita Tam, Mareike Möller, Runpeng Luo, Zhenyan Luo, Ashley Jones, Sambasivam Periyannan, John P. Rathjen, Benjamin Schwessinger

**Affiliations:** 1 Research School of Biology, Australian National University, Canberra ACT 2601, Australia;; 2 Commonwealth Scientific and Industrial Research Organisation Agriculture and Food, Canberra ACT 2601, Australia;; 3 School of Agriculture and Environmental Science, Centre for Crop Health, University of Southern Queensland, Toowoomba, Queensland 4350, Australia

## Abstract

Phased telomere-to-telomere (T2T) genome assemblies are revolutionizing our understanding of long-hidden genome biology “dark matter” such as centromeres, rDNA repeats, inter-haplotype variation, and allele-specific expression (ASE), yet insights into dikaryotic fungi that separate their haploid genomes into distinct nuclei are limited. Here, we explore the impact of dikaryotism on the genome biology of a long-term asexual clone of the wheat pathogenic fungus *Puccinia striiformis* f. sp. *tritici*. We use Oxford Nanopore Technologies (ONT) duplex sequencing combined with Hi-C to generate a T2T nuclear-phased assembly with >99.999% consensus accuracy. We show that this fungus has large regional centromeres enriched in LTR retrotransposons, with a single centromeric dip in methylation that suggests one kinetochore attachment site per chromosome. The centromeres of homologous chromosomes are most often highly diverse in sequence, and kinetochore attachment sites are not always positionally conserved. Each nucleus carries a unique array of rDNAs with more than 200 copies that harbor nucleus-specific sequence variations. The inter-haplotype diversity between the two nuclear genomes is shaped by large-scale structural variations linked to transposable elements. ONT long-read cDNA analysis across dormancy and distinct host infection conditions revealed pervasive ASE for ∼20% of the heterozygous genes. Genes encoding secreted proteins, including putative virulence effectors, are significantly enriched in ASE genes that appear to be linked to elevated CpG gene body methylation of the lower-expressed allele. This suggests that epigenetically regulated ASE is likely a previously overlooked mechanism facilitating plant infection. Overall, our study reveals how dikaryotism uniquely shapes key eukaryotic genome features.

Telomere-to-telomere (T2T) and haplotype-resolved genome assemblies have become the norm in eukaryotic genomics with advances in long-read sequencing technologies. Complete genome assemblies are fundamental for addressing key questions in genome biology that were previously hidden in the “dark matter” of genomes. Key breakthroughs have revolved around centromeres and the embedded kinetochore attachment sites pivotal for understanding karyotype diversity and evolution (Logsdon et al. 2024; Mastrorosa et al. 2024), as well as the notoriously repetitive ribosomal DNA (rDNA) arrays whose sequences have only been recently completed in humans (Nurk et al. 2022) and *Arabidopsis* (Fultz et al. 2023). Full haplotype resolution enables precise characterization of inter-haplotype variations predominantly shaped by heterozygosity, structural rearrangements, and transposable element (TE) movements (Gluck-Thaler et al. 2022; Hartmann 2022; Ferguson et al. 2024). Further, the complete picture of allelic information facilitates robust assessment of allele-specific expression (ASE) uncovering the underlying variations in *cis*-regulatory elements and epigenetic regulation, which can have important implications on phenotypic variability, as well established in mammals and plants (e.g., Shao et al. 2019; Cleary and Seoighe 2021; St. Pierre et al. 2022; Tian et al. 2022; Shi et al. 2024).

Although there have been important novel insights into diploid and polyploid genome organization, especially in plants (e.g., Belser et al. 2021; Hu et al. 2021; Sun et al. 2022), less is known about the fungi-specific dikaryotic state in which two haploid genomes are contained in separate nuclei in the same cytoplasm and propagated in a coordinated manner during cell division (Anderson and Kohn 2014; Li et al. 2019; Sperschneider et al. 2023a,b). Dikaryotism is highly successful, with an estimated 400,000 taxa in the fungal subphylum Basidiomycota relying on it for significant time periods, for example, during fruiting body formation in mushrooms and infection processes in rusts and smuts (Schmidt-Dannert 2016; James et al. 2020). Early partially phased assemblies of rust fungi genomes indicated high levels of heterozygosity and the presence/absence polymorphisms between the nuclear genomes; however, they lacked the resolution to identify individual haplotypes (Cuomo et al. 2017; Schwessinger et al. 2018, 2020; Vasquez-Gross et al. 2020). The latest fully haplotype-phased and nuclear-assigned T2T genomes have provided the first insights into organization of genes involved in mating behavior and contributed to our understanding of reproductive mechanisms generating novel genetic diversity (Schwessinger et al. 2022; Li et al. 2023a; Luo et al. 2024). This includes the somatic exchanges of nuclei between asexual rust lineages that are adapted to the infection of cereals (Sperschneider et al. 2023a; Henningsen et al. 2024), and sexual recombination in permissive hosts (Rodriguez-Algaba et al. 2022; Wang et al. 2022; Du et al. 2023). Such genetic reshuffling produces novel allele combinations of so-called avirulence (*Avr*) genes that encode secreted effector proteins essential for host infection. Rust effectors are under strong selection pressure to diversify into nonrecognized virulence alleles because they can be recognized by cognate immune receptors encoded by wheat resistance genes (Chen et al. 2017; Salcedo et al. 2017; Ortiz et al. 2022). This is necessary to escape the plant immune system and to confer the ability to infect new host varieties.

Recent progress in heterokaryotic fungi genomics, such as in arbuscular mycorrhizal fungi, has revealed extensive nuclear genome variations in structural and gene content (Li et al. 2019; Sperschneider et al. 2023b), as well as nuclear-level transcriptomic and epigenetic differences similar to findings in button mushroom *Agaricus bisporus* (Gehrmann et al. 2018), although key questions remain around the impact of dikaryotic genome organization on the evolution of centromeres, rDNA repeats, detailed inter-haplotype variations, ASE and methylation differences at locus resolution. Here, we use a long-term asexual clone of the wheat stripe rust fungus *Puccinia striiformis* f. sp. *tritici* (*Pst*) dating back at least 80 years to address these questions in a globally important wheat pathogen (Wellings 2007; Thach et al. 2016; Schwessinger et al. 2018). We leveraged high-accuracy Oxford Nanopore Technologies (ONT) duplex long reads to generate the first ONT-based T2T, fully nuclear-phased genome assembly for a dikaryotic fungus. We combined this with comprehensive ONT long-read cDNA data sets sampled during dormancy and host pathogenesis for high-quality gene annotations and differential expression analysis at gene- and allele-specific levels. Our study sheds new light onto the genome biology and adaptive evolutionary potential of rust fungi, with important implications for managing their agricultural impacts.

## Results

### A T2T nuclear-phased genome assembly of *Pst*104E based on high-quality ONT long-read sequencing

**Figure 1.**
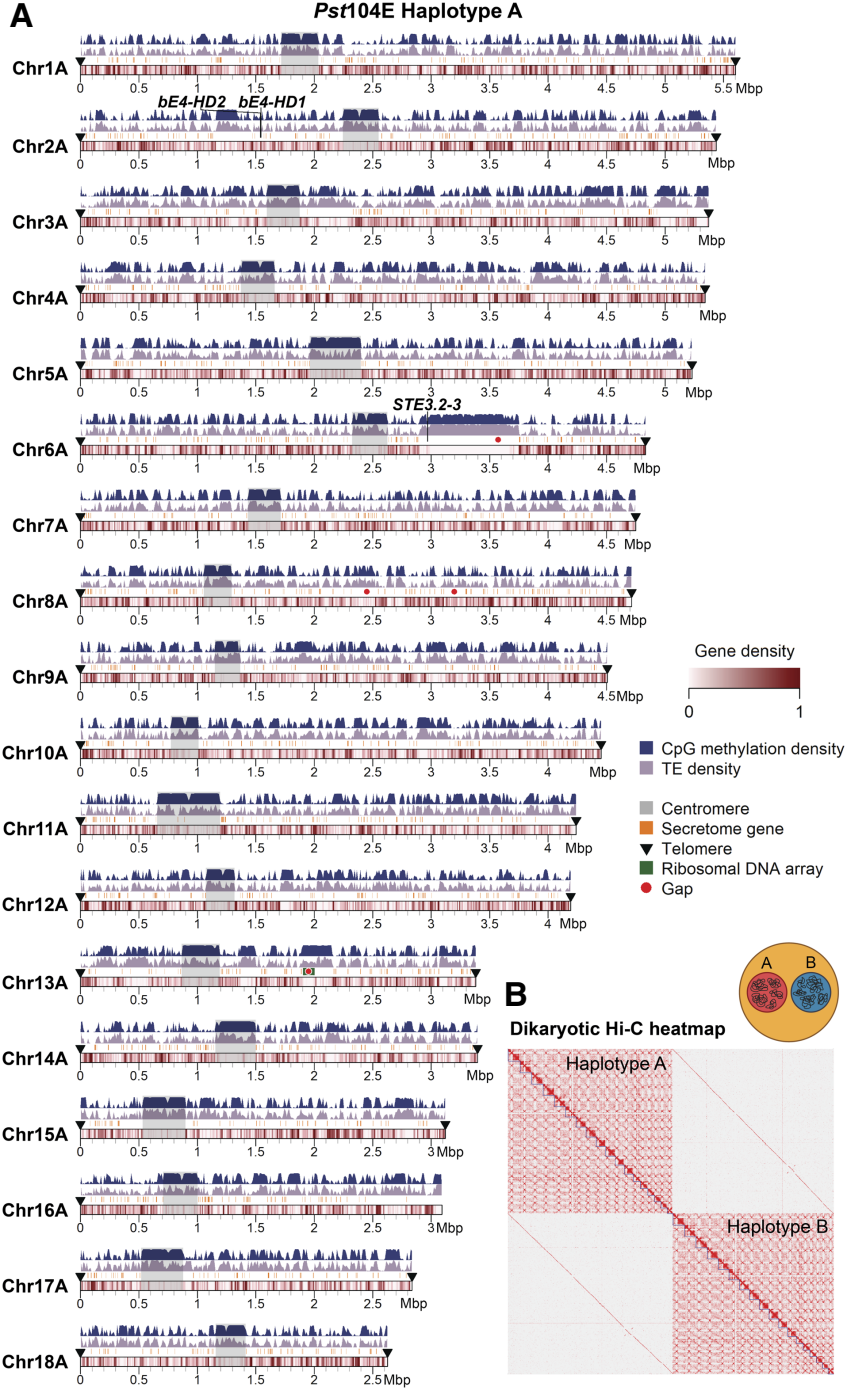
A nuclear-phased, chromosome-scale genome assembly of the dikaryotic fungus *Puccinia striiformis* f. sp. *tritici* (*Pst*) isolate 104E. (*A*) Karyoplot of the 18 chromosomes of haplotype A, showing CpG methylation and transposable element (TE) density as peaks, and gene density as heatmaps within chromosome ideograms (10 kbp sliding windows). Locations of centromeres, telomeres, secretome genes, the ribosomal DNA (rDNA) array, and assembly gaps are annotated as per the legend. The mating type loci are labeled in black text *above* the corresponding chromosomes. (*B*) Hi-C contact heatmap of the full dikaryotic genome assembly consisting of 36 chromosomes. The two nuclear haplotypes A and B display a clear signal of spatial separation.

We generated a dikaryotic *Pst* genome assembly for a representative isolate of the Australian founder pathotype 104E137A− (abbreviated as *Pst*104E), which belongs to the long-term asexual *Pst*S0 lineage (Wellings 2007; Schwessinger et al. 2018, 2020). We assembled ONT duplex reads combined with ultralong simplex reads and Hi-C (Supplemental Table S1), followed by scaffolding and manual curation. This resulted in 36 chromosome assemblies corresponding to the 18 homologous pairs, which we sorted by average length of both haplotypes (Chr 1 to Chr 18) (Fig. 1A; Table 1; Supplemental Fig. S1). Of these, 35 were assembled T2T, and each telomere had about 43 repeats on average, consistent with other basidiomycetes (Ramírez Nasto et al. 2011; Schwessinger et al. 2020; Sperschneider et al. 2021). The Hi-C contact heatmap clearly grouped the homologous chromosomes into two nuclear complements as expected for dikaryons (Fig. 1B). Five remaining gaps were found at repetitive regions, for example, a ∼650 kbp TE-rich region on Chr 6A near the previously identified mating type *PR* locus (Supplemental Fig. S2; Luo et al. 2024), as well as two rDNA arrays on Chr 13A and Chr 13B (Supplemental Fig. S3).

**Table 1.** Summary of assembly statistics and quality metrics of the ONT duplex genome assembly of *Pst*104E

Statistic	Full dikaryotic	Haplotype A	Haplotype B
Assembly size (bp)	152,328,638	77,128,783	75,199,855
No. of T2T chromosomes	35/36	17/18	18/18
N50 (bp)	4,614,849	4,713,886	4,606,998
% GC content	44.41	44.41	44.42
% TE content	44.51 (68 Mbp)	45.16 (35 Mbp)	43.87 (33 Mbp)
No. of gaps	5	4	1
% complete BUSCOs	92.6	91.9	91.4
No. of heterozygous SNPs/Mbp	6.8	6.9	6.7
No. of homozygous SNPs/Mbp	1.1	1.3	0.8
LAI	18.3	27.2	24.8
CRAQ R-AQI	94.7	94.3	95.1
CRAQ S-AQI	97.4	97.4	97.4

We evaluated the quality of the final curated assembly. The full assembly yielded 92.6% of complete BUSCOs (Manni et al. 2021). We used the long terminal repeat (LTR) assembly index (LAI) (Ou et al. 2018) to assess contiguity at repetitive LTR retrotransposons. Haplotype A and B assemblies each received LAI score of 27.2 and 24.8, classifying them to the highest rank based on well-assembled repeats. The per-base accuracy Phred score was 57.4 corresponding to >99.999% consensus accuracy, in line with recent Pacific Biosciences (PacBio) HiFi assemblies (Li et al. 2023a; Sperschneider et al. 2023a; Wang et al. 2024). CRAQ (Li et al. 2023b) was used to detect clipped alignments indicative of regional and structural errors that could be computed into R/S–assembly quality indices (AQIs). Our assembly achieved R/S-AQIs of 94.7 and 97.4, both meeting reference quality. Rare residual errors were reflected by the low density of SNPs detected as homozygous (1.1/Mbp, 161 total) and heterozygous (6.8/Mbp, 1038 total).

Analysis of the mapping read depth supported full haplotype phasing with a single peak corresponding to the expected haploid 1× depth (Supplemental Fig. S4). To detect putative phase switch errors, we quantified Hi-C paired alignments within and between haplotypes. About 99.2% of the Hi-C mappings were between chromosomes contained within one nucleus (within-haplotype links), with 0.8% linking chromosomes contained in separate nuclei (cross-haplotype links) (Table 2; Supplemental Fig. S5). These results demonstrate a highly complete and accurate nuclear-phased genome assembly of *Pst*104E.

**Table 2.** Hi-C contact statistics confirming phasing correctness of the *Pst*104E genome assembly

Types of Hi-C contacts (MAPQ ≥ 20)	Counts
Total Hi-C links	1,126,566
Within-haplotype links	1,117,107 (99.2%)
*cis*-chromosome	
Haplotype A	479,583 (42.6%)
Haplotype B	467,937 (41.5%)
*trans*-chromosome	
Haplotype A	85,667 (7.6%)
Haplotype B	83,920 (7.5%)
Cross-haplotype links	9459 (0.8%)

### ONT cDNA sequencing enables high-quality evidence-guided gene annotations

We aimed to improve on current fungal gene annotations by incorporating extensive long-read cDNA sequencing data sets (Pardo-Palacios et al. 2024). We generated a detailed time course of ONT direct cDNA data sets for *Pst*104E gene annotation. The transcripts were sampled from six conditions with four replicates each (Supplemental Table S2). These included ungerminated (UG) urediniospores as the dormancy control and infected wheat leaf tissues at 4, 6, 8, 10, and 12 days post infection (dpi). Principal component analysis (PCA) demonstrated clear clustering of the technical replicates of each sample, with separation of samples representing dormancy (UG) and macroscopically asymptomatic (4, 6, and 8 dpi) and symptomatic (10 and 12 dpi) stages (Supplemental Fig. S6). We also complemented gene annotation with multiple publicly available Illumina RNA-seq data sets (Supplemental Table S3; Dobon et al. 2016; Schwessinger et al. 2018; Zhao et al. 2021). In total, we annotated 15,142 protein-coding genes on haplotype A and 14,938 on haplotype B, improving the complete BUSCO score to 94.8%. Functional annotation identified ∼15% of the gene models that encode secreted proteins without predicted transmembrane domain.

ONT direct cDNA sequencing has demonstrated quantitative power consistent with short-read RNA-seq, making it suitable for differential expression analysis (Sessegolo et al. 2019; Grünberger et al. 2022; Pardo-Palacios et al. 2024). We performed differential expression analysis with our ONT cDNA data sets to identify candidate *Avr* effector genes. We searched for secretome genes that are upregulated early in wheat infection (4, 6, and 8 dpi) relative to UG, as their functions likely correlate with pathogenesis. A total of 1318 secretome genes were found to be upregulated early during infection. These were shortlisted to hemizygous genes (single copy) for reduced functional redundancy, resulting in 97 high-priority candidates for future functional validation (Supplemental Table S4).

### TE annotations

TEs are major components of genomes of many basidiomycetes (Castanera et al. 2017; Corre et al. 2025). TE annotations for our *Pst*104E assembly revealed that both haploid genomes shared similar TE content and composition, covering 44.51% of the genome space (Table 1). This was consistent with findings for other published *Pst* genomes (Zheng et al. 2013; Schwessinger et al. 2018, 2022). Class I (retrotransposons) and Class II (DNA transposons) accounted for 15% and 18.7%, respectively, of the genome (Supplemental Table S5). LTRs comprised the most abundant retrotransposons, predominated by the Ty3/Gypsy. Terminal inverted repeats (TIRs) represented the majority of DNA transposons.

### *Pst* centromeres are highly diverse and enriched with LTR retrotransposons

**Figure 2.**
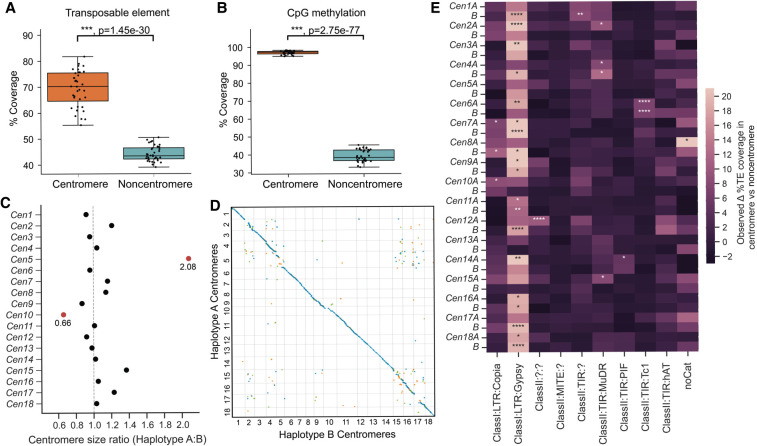
*Pst*104E centromeres are highly diverse with haplotype-specific sequences and are enriched in retrotransposons. (*A*) Percentage of TE coverage in centromere and noncentromere regions of *Pst*104E. Each dot represents one of the 36 chromosomes. Student's *t*-test: (***) *P* < 0.001. (*B*) Percentage of coverage of methylated CpG sites in centromere and noncentromere regions of *Pst*104E. Each dot represents one of the 36 *Pst*104E chromosomes. Student's *t*-test: (***) *P* < 0.001. (*C*) Size ratio of haplotype A centromeres compared to haplotype B centromeres. Outlier ratios exceeding 1.5 times the interquartile range are highlighted in red. (*D*) Pairwise alignment dotplot between centromeres of haplotypes A and B. Only alignment blocks >100 bp with a minimum of 90% sequence identity are shown. Each color denotes an alignment type: blue, unique forward alignments; green, unique reverse alignments; and orange, repetitive alignments. (*E*) Enrichment of TE superfamilies within centromeres compared with noncentromere regions. Statistical significance was assessed using permutation tests on each chromosome. Only abundant TE superfamilies with >1% of total genome coverage are shown. The color scalebar denotes the test statistic, defined as the observed difference in the percentage of TE coverage within and outside centromeres. *P*-values represent the proportion of permuted values equal to or more extreme than observed. FDR < 5% was applied to correct for multiple testing: (*) *P* < 0.05, (**) *P* < 0.01, (****) *P* < 0.0001.

We set out to identify *Pst*104E's centromeric regions and analyzed their sequence composition. Basidiomycete centromeres are often characterized by hypermethylated TE-rich and gene-poor regions (Guin et al. 2020). We estimated their positions from the Hi-C heatmap based on the strong inter-chromosomal contact peaks (“bowtie” shapes) caused by *Rabl* centromere clustering (Fig. 1B; Varoquaux et al. 2015; Muller et al. 2019). These interaction sites were enriched for TEs and overlapped with gene-sparse regions spanning hundreds of kilobases (Figs. 1A, 2A). Using ONT-derived methylation data, we found substantially more methylated CpGs within all 36 inferred centromeric regions, *Cen1A* to *Cen18B* (94.9%–98.3%), than in noncentromeric regions (32.9%–45.3%) (Fig. 2B).

*Pst*104E centromere sizes varied approximately 2.5-fold between 210 and 538 kbp (mean 304 kbp) (Supplemental Table S6), categorizing them as “large regional” centromeres that are known to support multiple spindle microtubule attachments (Bodor et al. 2014; Yadav et al. 2018a). Most homologous chromosomes shared similar centromere lengths with differences under 1.4-fold, whereas *Cen5A* stood out being double the length of *Cen5B* (Fig. 2C). Pairwise alignments of centromeric regions revealed varying levels of macrocollinearity between haplotypes, ranging from almost complete (e.g., *Cen3*, *-12*, -*13*, and -*18*) to negligible synteny (e.g., *Cen2*, *-5*, *-15*, and -*17*) (Fig. 2D). Given the high TE density, such a diverse range in centromeric sequence conservation prompted us to examine their TE composition in search of elements possibly linked to centromere function.

We conducted permutation tests to determine the enrichment of abundant TE superfamilies within each *Pst* centromere compared with noncentromeric regions (Fig. 2E; Supplemental Table S7). Most centromeres (25 out of 36) were found to be significantly enriched for one to two TE superfamilies that belonged to retro- and/or DNA transposons (Fig. 2E). Of these, 19 centromeres were significantly enriched for Ty3/Gypsy LTRs, occasionally cocolonized by TIRs. Given the balanced representation of retro- and DNA transposons throughout the genome, this finding suggests that retrotransposons might have a more prominent role in *Pst* centromere formation than DNA transposons.

### Centromeres contain a single putative kinetochore attachment site

**Figure 3.**
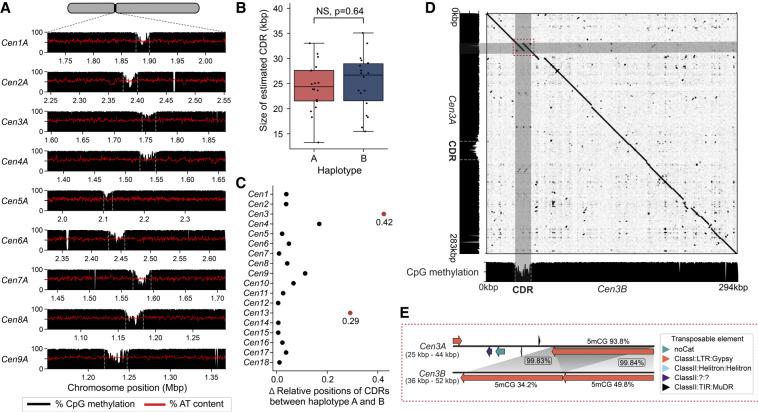
Analysis of centromere dip regions (CDRs) within *Pst*104E centromeres suggest a single putative kinetochore attachment site per chromosome. (*A*) CpG methylation profiles (black histograms) and percentage of AT content (red lines) across *Pst*104E centromeres *Cen1A* to *Cen9A* as examples. A consistent methylation depletion valley with a mean size of ∼24.8 kbp was observed throughout all centromeres, indicating CDR signals (marked by gray dotted line). (*B*) Estimated sizes of the CDRs for each chromosome in haplotype A and B. Student's *t*-test: (NS) *P* > 0.05. (*C*) Differences in the relative positions of CDRs between haplotypes for each homologous chromosome pair. Differences that exceed 0.2 are highlighted in red. (*D*) Sequence alignment dotplot between *Cen3A* and *Cen3B* and their respective CpG methylation profiles (black histograms). The dotted red box highlights sequence divergence between *Cen3A* and *Cen3B* corresponding to the *Cen3B* CDR. (*E*) Detailed synteny analysis of the region highlighted in *D*. Long orange arrows indicate copies of hapB-B-G1437-Map9 belonging to the Ty3/Gypsy LTR retrotransposon superfamily. Gray shading indicates homologous sequences with percentage identity shown in boxes. Numbers *above* and *below* the orange arrows indicate the respective percentage of methylated CpGs.

It is unclear if rust fungi have one or multiple kinetochore attachment sites given they have “large regional” centromeres (Fig. 2; Yadav et al. 2018a; Sperschneider et al. 2021). Kinetochores typically assemble at a hypomethylated stretch of DNA embedded within the centromere termed the “centromere dip region” (CDR), which is marked by the centromere-specific histone variant CENP-A (Akiyoshi 2019; Sundararajan and Straight 2022; Logsdon et al. 2024). We examined the CpG methylation pattern along the *Pst*104E centromeres to locate CDRs as a qualitative proxy for potential kinetochore sites. Throughout all centromeres, we consistently observed a single methylation depletion “valley” spanning 24.8 kbp on average, which is characteristic of CDR (Fig. 3A; Supplemental Fig. S7; Supplemental Table S6). The CDR lengths were similar between haplotypes (Fig. 3B). The positioning of CDRs were generally conserved (Figs. 3C, 2D). The two notable exceptions were *Cen3* and *Cen13*, whose CDR haplotypes were placed further than 20% of their centromere lengths apart (Fig. 3C).

We next investigated whether the CDR signals were linked to increased AT content as reported for other fungi (Yadav et al. 2018a; Sankaranarayanan et al. 2020; Narayanan et al. 2024). No differences in AT content were detected when comparing CDRs with centromeric or noncentromeric regions (Supplemental Fig. S8). The only exception was *Cen3B* CDR, which has an elevated AT content of 59.8% compared with the rest of the centromere and the overall genome average of 55.6%. Close investigation revealed two nearly identical AT-rich copies of a Ty3/Gypsy retrotransposon family (hapB-B-G1437-Map9), together covering 91% of *Cen3B* CDR. Sequence alignment revealed that the corresponding syntenic region on *Cen3A* contained only one copy of this TE family, which was not involved in CDR formation (Fig. 3D). Consistently, this single copy on *Cen3A* was highly methylated on its CpG sites (93.8%), whereas the two TE copies on *Cen3B* CDR were lowly methylated (34.7% and 49.8%) (Fig. 3E). This was despite >99.5% identity shared by the three TE copies. These results highlight that centromere and kinetochore attachment site formation is not solely driven by primary DNA sequence composition.

### Nucleus-specific variations in the rDNA arrays

**Figure 4.**
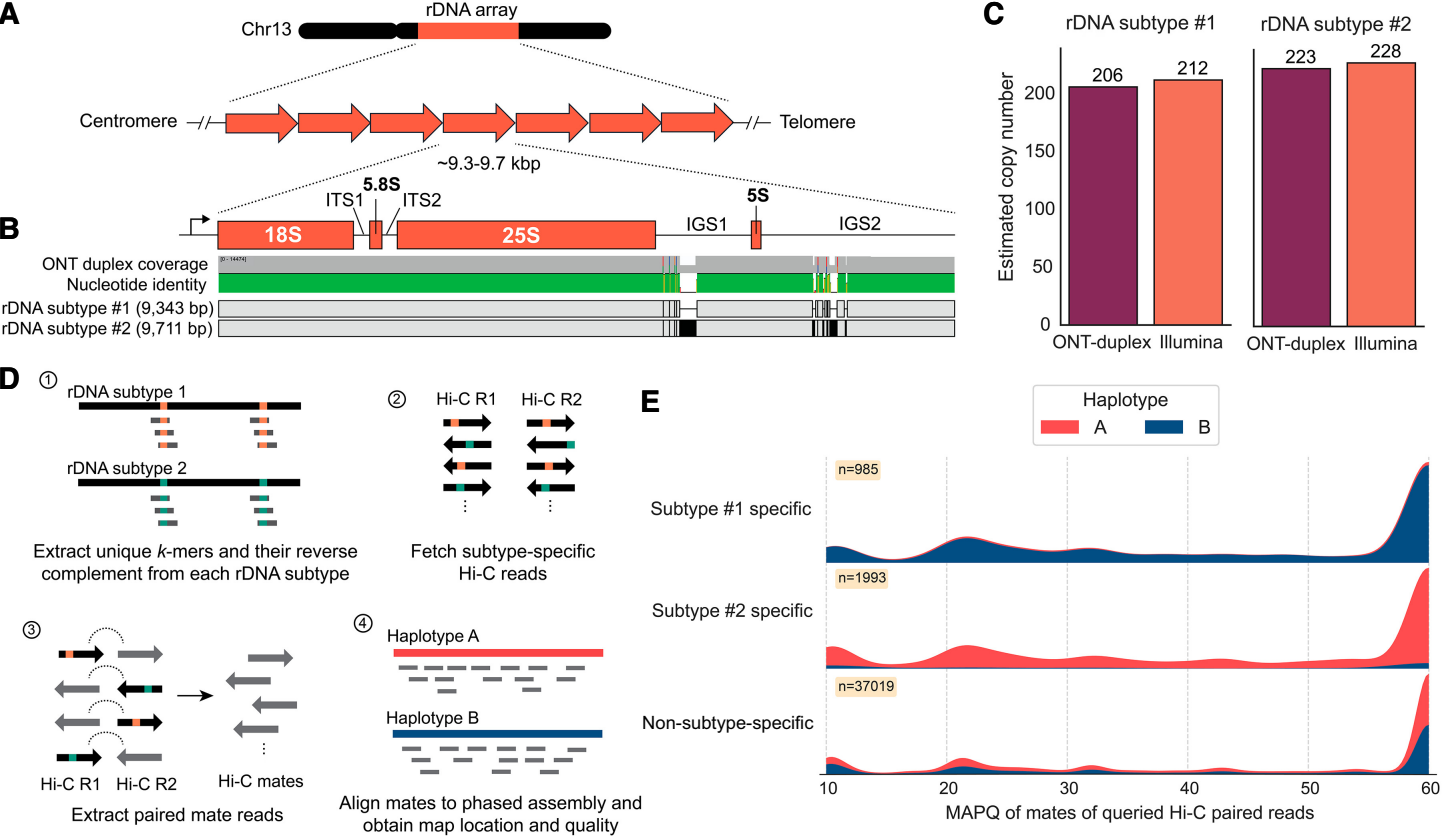
The two dikaryotic nuclear haplotypes of *Pst*104E harbor distinct rDNA subtypes. (*A*) Schematic view of the rDNA tandem repeat array located on *Pst*104E Chr 13. (*B*) Diagram of a single canonical rDNA unit of *Pst*104E containing the transcription start site in the 5′ external transcribed spacer, the catalytic rRNA genes (18S, 5.8S, 25S, and 5S), two internal transcribed spacers (ITS1 and ITS2), and two intergenic spacers (IGS1 and IGS2). The alignment shows two dominant rDNA subtypes (1 and 2), which differ by SNPs and structural variations in both IGS. (*C*) Estimated copy number of rDNA subtypes 1 and 2 based on ONT duplex and Illumina read data sets. (*D*) Workflow of our rDNA Hi-C analysis designed to determine the physical location of the two dominant rDNA subtypes in each nuclear haplotype. (*E*) Mapping quality (MAPQ) distributions of Hi-C reads whose paired mates contained rDNA subtype-specific or non-subtype-specific 31-mers, categorized by the nuclear haplotype they mapped against.

We investigated the rDNA composition in *Pst*104E to better understand its dynamics in the context of a dikaryotic genome with physical separation of the haploid genomes. *Pst*104E has a single rDNA cluster per nuclear genome on the q-arm of Chr 13 (Fig. 1A). Both haplotypes were incompletely assembled with a gap at the rDNA cluster, indicating an underrepresentation of this complex locus (Supplemental Fig. S3). The assembled rDNA copies were oriented with transcription directed away from the centromere and toward the telomere (Fig. 4A). We reconstructed the canonical rDNA repeat (see Methods), which included the 45S transcription unit (18S, 5.8S and 25S rRNA genes) separated by two internal transcribed spacers (ITS1 and ITS2), followed by two intergenic spacers (IGS1 and IGS2) and a 5S rRNA gene that is barely transcribed in between (Fig. 4B; Supplemental Fig. S9). We defined 18S and IGS2 as the start and end of an rDNA unit for the subsequent analysis.

Detailed analyses of sequencing reads revealed two major rDNA subtypes (1 and 2) supported by substantial read coverage at near-equal frequencies. The estimated copy number ranged from 206 to 212 copies for rDNA subtype 1 and 223 to 228 copies for subtype 2, totaling about 434 copies (Fig. 4C). The two subtypes were 9343 bp and 9711 bp in length, with polymorphisms contained in the repeats nested within IGS1 and IGS2 (96.1% sequence identity) (Supplemental Fig. S10). We also identified 12 variants of the two subtypes (1.1–1.9 and 2.1–2.3) based on low-frequency SNP analysis (Supplemental Fig. S11; Supplemental Table S8). Most of these SNPs occurred in the 18S gene. The subtype variants 1.1–1.9 and 2.1–2.3 collectively accounted for ∼23% of the total copy number, suggesting incomplete rDNA homogenization (Supplemental Table S8).

Given the long clonal history of *Pst*104E (Schwessinger et al. 2020), the individual haplotypes are expected to have been stably inherited in separate nuclei without karyogamy or meiotic crossovers. We therefore hypothesized that the sequence variations in the two dominant rDNA subtypes might be nucleus specific. To test this, we took advantage of Hi-C read pairs that contained rDNA subtype-specific *k*-mers and asked where the alternate read mate pair mapped (Fig. 4D). About 94.3% of mates associated with rDNA subtype 1 mapped to haplotype B, whereas 90.2% of those associated with subtype 2 mapped to haplotype A (Fig. 4E); the remaining cross-haplotype links were likely technical noises owing to some less informative subtype-specific *k*-mers (such as those derived from the IGS2 minisatellite-like repeats) and sequencing artifacts. The control procedure, which involved aligning subtype-nonspecific rDNA Hi-C read pairs, showed a near-equal proportion of Hi-C mates mapped against each haplotype. Together, this implies that each rDNA subtype was associated with a different haplotype and that each nuclear genome contains its own major rDNA subtype array.

### Large-scale structural variations driven by TEs shape inter-haplotype diversity

**Figure 5.**
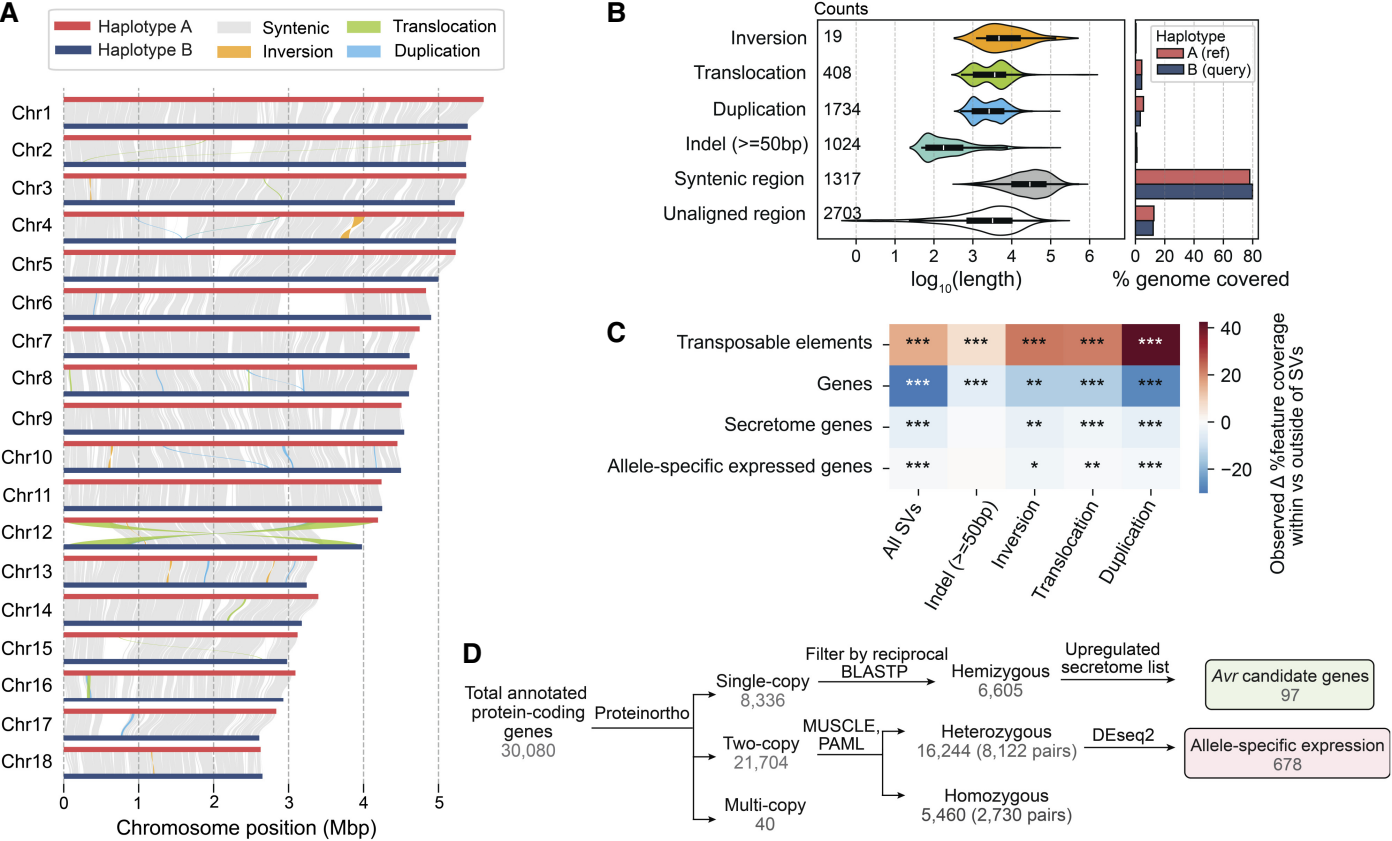
*Pst*104E*’s* inter-haplotype variation is shaped by large-scale structural variation (SV) and TEs. (*A*) Synteny and structural rearrangements between chromosome pairs of the two nuclear haplotypes, with A as reference and B as query. (*B*) Length distribution (*left*) of different SV types, including syntenic and unaligned regions. Counts of each SV type are shown. The bar chart (*right*) represents the total genome length covered by each sequence category. (*C*) Enrichment (red) and depletion (blue) of different genomic features (TEs and genes) within SVs including ±2 kbp flanking regions tested using permutations. The color scale denotes the observed difference in the percentage of coverage of a genomic feature located within and outside of a given set of SVs. *P*-values indicate the proportion of permuted coverage values less than, equal to, or greater than the observed: (*) *P* < 0.05, (**) *P*<0.01, (***) *P*<0.001, FDR-corrected. (*D*) Flowchart summarizing the classification of hemizygous, heterozygous, and homozygous protein-coding genes and their further categorization. Hemizygous genes were intersected with secretome genes upregulated during infection time points (4, 6, or 8 dpi) to shortlist *Avr* candidates. Heterozygous biallelic genes were used for allele-specific expression (ASE) analysis.

The phased *Pst*104E assembly allowed us to assess its inter-haplotype structural variations (SVs). The haplotypes were 78.8% syntenic (Fig. 5A), with 60.2 Mbp of haplotype A (78.0%) and 60.1 Mbp of haplotype B (79.6%) identified as highly continuous syntenic blocks (Fig. 5B). All identified SV types (duplications, large indels, translocations, and inversions) collectively occupied ∼11% and 9%, respectively, of the total lengths of haplotypes A and B (Fig. 5B; Supplemental Table S9). About 10% of each haplotype's sequence was not alignable and was therefore hemizygous. Syntenic regions had the largest average length, with most ranging 10–100 kb. The different SV types had various length distributions mostly centered at ∼1–10 kb (Fig. 5B). We used permutations to test if the SVs and their 2 kbp flanking regions were enriched for specific genomic features. This revealed a significant enrichment of TEs across all analyzed SV types, especially duplications, possibly owing to the replicative mechanism of retrotransposons (Fig. 5C; Supplemental Table S10). In contrast, protein-coding genes were depleted at SVs.

Next, we assessed the impact of inter-haplotype variation on genes by analyzing their sequence conservation and synteny (Fig. 5D). We robustly identified 3391 and 3214 hemizygous genes unique to either haplotype A or B, respectively. A total of 21,744 genes shared at least one homolog in the alternative haplotype. We analyzed one-to-one gene pairs and performed codon-aware alignment to compute their divergence values at synonymous and nonsynonymous sites (Supplemental Table S11), identifying 2730 homozygous pairs. The remaining 8122 displayed divergence greater than zero and were therefore defined as heterozygous biallelic pairs.

### ASE is correlated with gene body methylation and enriched for genes encoding effector/secreted proteins

We next investigated if any of the heterozygous biallelic genes displayed ASE in any condition using our ONT cDNA data sets.

**Figure 6.**
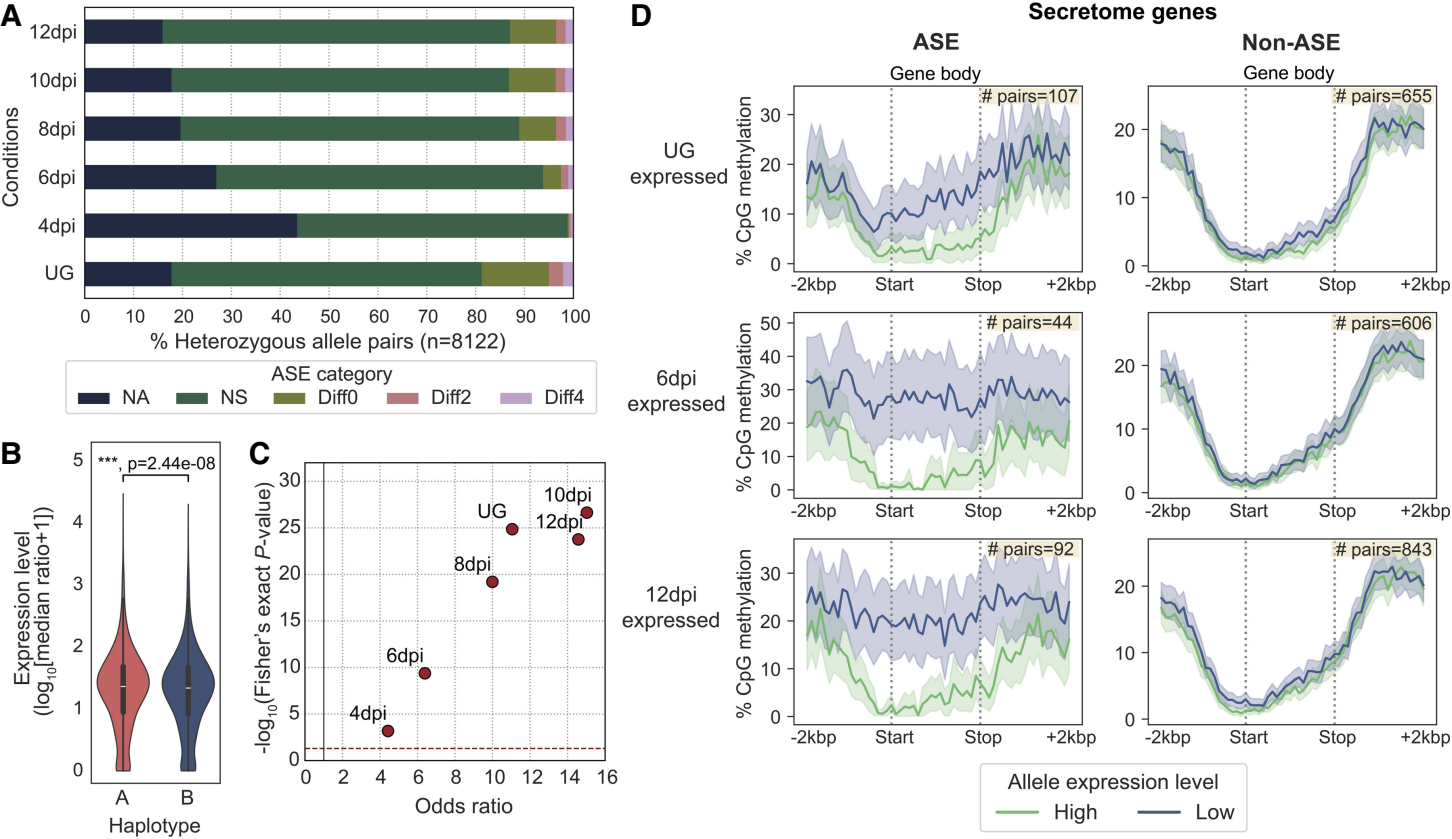
ASE is prevalent among secretome genes and correlates with gene methylation patterns. (*A*) The five ASE categories detected across six different transcript sampling conditions. Allele pairs displaying absolute log_2_ fold changes greater than two (Diff2 and Diff4) in at least one condition were defined as ASE in subsequent analysis. (*B*) Expression levels of alleles belonging to the two nuclear haplotypes A and B. Mann–Whitney *U* test: (***) *P* < 0.001. (*C*) Odds ratios and log_10_ *P*-values from Fisher's exact tests comparing the proportion of ASE genes among secretome genes and evolutionarily conserved BUSCOs. Each red dot represents a transcript sampling conditions as labeled. Dotted red and gray lines highlight cut-offs matching the null hypothesis of no difference between the two gene groups. (*D*) Distribution of the percentage of CpG methylation (sampled at UG) across ASE or non-ASE secretome gene bodies (here defined as start to stop codon) including ±2 kbp flanking regions. Solid lines represent mean percentage of CpG methylation; shaded areas represent 95% bootstrapping confidence intervals. Yellow *inset* shows the number of allele pairs included.

We first tested if there was an overall gene expression bias at the individual nuclear haplotype level. Both haplotypes displayed balanced expression without evidence of nuclear dominance (Supplemental Fig. S12); however, an expression bias for haplotype A was detected when considering only the heterozygous biallelic genes (Fig. 6B).

We compared the transcript abundance for each heterozygous allele pair to determine its ASE across all six tested conditions (Fig. 6A; Supplemental Table S12). We classified the ASE status using the following criteria (Shi et al. 2024): (1) no observed expression or no unambiguous transcript mapping at both alleles (NA), (2) no significant difference between alleles with false-discovery rate (FDR) adjusted *P*-value > 0.05 (NS), and (3) significant difference between alleles with adjusted *P*-value < 0.05, indicating differential ASE. The differential ASE pairs were further classified based on log_2_ fold change (LFC): weak ASE, |LFC| < 2 (Diff0); moderate ASE, 2 ≤ |LFC| < 4 (Diff2); and strong ASE, |LFC| ≥ 4 (Diff4). Although most heterozygous genes showed no evidence of ASE, a substantial proportion exhibited ASE in each condition. The UG stage had most ASE pairs (18.7%), followed by 8 to 12 dpi, which maintained stable ASE rates (11.1%–13.2%). Early infection at 4 dpi had the fewest (1.2%) ASE pairs, potentially owing to the low fungal biomass sampled. To reduce noise in the subsequent ASE analyses, we applied a |LFC| threshold of two to define the ASE set (Diff2 and Diff4), whereas all other categories were defined as non-ASE for the remainder of this study. Intersection analysis identified 678 allele pairs (8.3%) that were assigned to Diff2 or Diff4 ASE in at least one condition (Supplemental Fig. S13).

Given that pathogenicity-related effectors typically undergo diversifying selection (Stukenbrock and McDonald 2009; Sperschneider et al. 2014), we hypothesized that ASE might be an additional pathway to drive virulence dynamics. We therefore asked whether secretome genes, including putative effectors, were overrepresented in each condition-specific ASE versus non-ASE set compared with those of evolutionarily conserved BUSCOs. A Fisher's exact test (FDR < 5%) for every condition consistently showed significant ASE enrichment for secretome genes relative to BUSCOs with odds ratios greater than one (Fig. 6C; Supplemental Table S13, see contingency tables).

We explored several epigenetic and genetic factors that potentially underlie the ASE of secretome genes. No association was found between ASE and SVs (Fig. 5C), TE occupancy, and allelic sequence divergence (Supplemental Figs. S14, S15; Supplemental Note). We compared the CpG methylation density between secretome alleles along their gene bodies (start to stop codon) and 2 kbp flanking sequences to include proximal *cis*-regulatory elements such as promoters. Significant methylation differences were observed around the start codon between ASE alleles across all in planta infection conditions compared with the non-ASE alleles (Fig. 6D; Supplemental Fig. S16). Higher-expressed alleles were found to be hypomethylated at the start codon windows (1.1%–2.5% CpGs methylated), whereas those of their lower-expressed counterparts were frequently more heavily methylated (19.2%–36.1% CpGs methylated). Non-ASE secretome genes showed no methylation difference between alleles, regardless of their expression levels. These data suggest that DNA methylation imbalance may be involved in secretome ASE.

## Discussion

Recent advances in long-read sequencing technologies have made T2T haplotype-phased genome assemblies the new gold standard for eukaryotes, including di- and heterokaryotic fungi such as rust fungi. Here, we report a fully nuclear-phased T2T genome assembly for *Pst*, the first reconstructed using high-accuracy ONT duplex sequencing. We show that our ONT-only T2T genome assembly is of comparable or superior quality to recently published PacBio HiFi-based haplotype-resolved T2T assemblies of other di- and heterokaryotic fungi (e.g., Sperschneider et al. 2023a; Henningsen et al. 2024; Wang et al. 2024). With the complete resolution of both nuclear haplotypes in our *Pst* assembly, we were able to uncover novel insights into the impact of dikaryotism on the genome biology of a long-term asexual clonal isolate of this fungus.

Our detailed analyses of *Pst* centromeres show that they adopt the classic *Rabl* configuration with clustering of heterochromatic centromeres (Xia et al. 2022; Torres et al. 2023). This arrangement allowed us to identify large regional centromeres from Hi-C contact hotspots that coincide with hypermethylated, gene-poor genomic signatures. Each centromere has a single potential kinetochore attachment site marked by a hypomethylated pocket known as the CDR (Logsdon and Eichler 2023; Logsdon et al. 2024; Mastrorosa et al. 2024), within otherwise fully CpG-methylated centromeres without signs of elevated AT content. Direct experimental evidence from CENP-A (CenH3) chromatin immunoprecipitation sequencing will be required to validate centromere localization. Our comparative inter-haplotype analysis revealed that *Pst* centromeres are highly variable in length and sequence, lacking characteristic motifs that define all centromeres. In most cases, the inferred kinetochore site appears to be consistently positioned in homologous chromosomes even considering highly divergent homologous centromeres, and where shifts occur, we did not detect an associated sequence presence/absence pattern. These findings point to the conclusion that formation of *Pst* centromeres is a sequence-independent process, consistent with observations in many other fungal regional centromeres (Roy and Sanyal 2011; Smith et al. 2012; Schotanus et al. 2015; Sperschneider et al. 2021; Cissé et al. 2024). Most but not all *Pst* centromeres are enriched for LTR retrotransposons, especially the Ty3/Gypsy superfamily. LTR-rich centromeres have also been reported for other pathogenic fungi with large regional type centromeres, such as the closely related stem rust fungus *Puccinia graminis* f. sp. *tritici* (Sperschneider et al. 2021), a human pathogenic yeast *Cryptococcus neoformans* (Yadav et al. 2018b), and an ascomycetous phytopathogen *Verticillium dahliae* (Seidl et al. 2020), but direct roles for LTR retrotransposons in centromere establishment remain unclear. In *C. neoformans*, the RNAi machinery and DNA methylation have been proposed as key epigenetic drivers for centromere identity via suppressing transposition and deleterious recombination among centromeric LTRs (Yadav et al. 2018b). However, the fact that the correlation with LTR enrichment does not hold in some *Pst* centromeres supports the idea that the LTR sequence itself does not define centromeres (Lynch et al. 2010; Guin et al. 2020). Rather, LTRs might be preferentially inserted owing to their high proliferative potential, which may, in turn, promote RNAi-directed silencing to reinforce centromere formation (Balzano and Giunta 2020). Further studies will be required to elucidate such links in *Pst*.

The dikaryotic configuration of *Pst* prompted us to explore its effect on intraspecific rDNA dynamics under strict clonality. Generally, rDNA tandem repeats are thought to undergo concerted evolution toward sequence homogenization via repeated homologous recombination, namely, unequal crossovers and gene conversion (Symonová 2019; Mullis et al. 2020; Garcia et al. 2024). Because *Pst*104E has genetically distinct nuclei, we hypothesized that its rDNA variants may persist or emerge within individual nuclei in the absence of meiotic exchange. Our results show that each nucleus of *Pst*104E carries a unique array with more than 200 repeats, predominated by an rDNA sequence subtype that harbors nucleus-specific variations within both IGS regions. A low proportion of these repeats have diversified through accumulating point mutations, and some appeared to have become fixed. We speculate that such nucleus-specific rDNA subtype homogeneity might be the consequence of compartmentalized concerted evolution owing to the individual inheritance of each nucleus over prolonged clonal history. The intra-array diversification, however, signifies a relaxation of concerted evolution, leading to incomplete homogenization within each nucleus (Xu et al. 2017; Wang et al. 2023). A possible explanation for this could be the reliance on limited nonmeiotic homologous recombination (e.g., intrachromosomal or between sister chromatids), which might less efficiently purge the newly spreading variants (Paloi et al. 2022). This parallels previous observations from homokaryotic (i.e., has genetically uniform nuclei) arbuscular mycorrhizal fungi strains (Serghi et al. 2021), which displayed extensive rDNA heterogeneity within each nucleus, consistent with their ancient clonality (Pawlowska and Taylor 2004; Lin et al. 2014). In the future, it will be interesting to survey intraspecific rDNA variations in rust isolates that have arisen via recent sexual recombination (Heitman et al. 2013; Wallen and Perlin 2018; Wang et al. 2022), in which we expect that divergent rDNA subtypes will be more evenly distributed among nuclei. Dikaryotism therefore presents an excellent opportunity for understanding the rates and dynamics of concerted evolution in fungi.

A fundamental question in genome biology is whether ASE could have functional consequences that lead to phenotypic variability (Cleary and Seoighe 2021; St. Pierre et al. 2022). Here, we show that ASE is pervasive in *Pst* and appears to be inversely related to gene body methylation, in which lower-expressed alleles display higher levels of CpG methylation. In *Pst*104E, ASE is overrepresented in secretome genes including putative effectors that could be involved in host pathogenesis. Therefore, ASE might present a novel transcriptomic regulatory mechanism to generate effector diversity beyond protein sequence variations (Chen et al. 2017; Salcedo et al. 2017; Ortiz et al. 2022). Expression-level polymorphisms between recognized and nonrecognized effector alleles may also explain virulence switching. For example, in *P. graminis* f. sp. *tritici*, a virulence allele of *AvrSr27* was expressed at a much lower level than its avirulence allele counterpart, yet when overexpressed in planta, recognition took place (Upadhyaya et al. 2021). Similar observations have been recently made in a common rust disease of maize caused by *Puccinia sorghi*. A lowly expressed virulence allele of *AvrRp1-*D, differing by only one amino acid from its avirulence counterpart, became recognized by the cognate resistance gene *Rp1-D* after co-overexpression in planta (Kim et al. 2024). Nonrecognition can be therefore be caused by low expression rather than an inability of the host and pathogen proteins to interact. Future studies will inform whether the ASE of these *P. graminis* f. sp. *tritici* and *P. sorghi* effector genes is linked to changes in gene body methylation. The role of epigenetic regulation of *Avr* gene expression was previously highlighted in the soybean pathogen *Phytophthora sojae*, in which the natural silencing of the avirulence gene *Avr3a* resulted in gain-of-virulence (Qutob et al. 2013; Hale et al. 2023). Such epigenetics-mediated ASE may offer a reversible means to selectively silence or “archive” avirulence alleles to escape immune recognition while retaining the unmutated gene.

One important limitation of our study is that our DNA methylation data only reflects dormancy. A previous study in *Zymoseptoria tritici* (Meile et al. 2020) demonstrated that chromatin remodeling via histone modifications, which are highly correlated with DNA methylation in fungi (Rose and Klose 2014; He et al. 2020), can occur during host pathogenesis to derepress effector expression. To extend our observations, we will need DNA methylation and histone modification data sampled from *Pst* undergoing pathogenesis to determine if chromatin dynamism underpins ASE in planta.

Flor's classic “gene-for-gene” hypothesis (Flor 1942) that has shaped our understanding of plant resistance to pathogens since the 1940s might be an oversimplification, and we may have to consider that differences in effector gene expression underlie disease outcomes in the field.

## Methods

Methods for *Pst*104E DNA and RNA extraction, ONT long-read genome and transcriptome sequencing, and Hi-C library sequencing are detailed in the Supplemental Methods.

### Genome assembly and quality evaluation

Filtered duplex (>10 kbp; Q30; 32×/haplotype) and simplex (>40 kbp; Q10; 117×/haplotype) reads were assembled using Verkko v1.3.1 (Rautiainen et al. 2023). The assembly was scaffolded with Hi-C using Juicer v2.0 (Durand et al. 2016b) and 3D-DNA v180114 (Dudchenko et al. 2017), followed by manual curation (Supplemental Methods). Each haplotype was quality-assessed using assembly statistics, BUSCO v5.5.0 (basidiomycota_odb10) (Manni et al. 2021), Merqury v1.3 (Rhie et al. 2020), and LAI (Ou et al. 2018; Ou and Jiang 2018). CRAQ v1.0.9 (Li et al. 2023b) was launched to compute R/S-AQI inferred from regional and structural errors from clipped alignments that may indicate misjoins. To evaluate phasing quality, HiC-Pro v3.1.0 (Servant et al. 2015) was used to generate contact matrices from Hi-C alignments (MAPQ ≥ 20), which were analyzed using scripts from GitHub (https://github.com/RunpengLuo/HiC-Analysis) to quantify *cis*- and *trans*-chromosome contacts.

### TE and gene annotations

TE and gene annotations were performed for each haplotype separately. We predicted and annotated TEs using the REPET pipeline v3.0 (Quesneville et al. 2005; Flutre et al. 2011). Prior to gene annotation, we filtered host RNA from all transcriptomic data sets by mapping them against the *Pst*104E assembly with minimap2 v2.26 (Li 2018) and retaining only the mappable reads. We then independently processed and assembled the Illumina RNA-seq and the ONT cDNA data sets to generate transcript evidence. For ONT cDNA, reads were trimmed with Porechop_ABI v0.5.0 (Bonenfant et al. 2022), aligned to the dikaryotic assembly with minimap2 in splice-aware mode (-ax splice -ub -G 3000 ‐‐secondary=no), and partitioned into haplotype sets. To identify transcript structures from noisy long reads reference-guided and annotation-free, we employed two long-read-tailored tools: StringTie2 (-L -s2 -m50) (Kovaka et al. 2019) for better single-exon transcript discovery and ESPRESSO v1.4.0 (ESPRESSO_S.pl -Q0) (Gao et al. 2023) for improved splice site detection. Transcript annotations were merged across all samples using StringTie2 (‐‐merge). Gene predictions and functional annotations were performed using funannotate v1.8.15 (https://github.com/nextgenusfs/funannotate). For details, see the Supplemental Methods.

### Differential expression analysis

Transcript abundance was quantified from ONT cDNA spliced alignments using Bambu v3.4.1 (Chen et al. 2023) with the isoform discovery mode disabled. The resulting count matrices were imported to DESeq2 v1.38.3 (Love et al. 2014) for analysis. PCA was performed on variance-stabilizing transformed read counts to visualize sample clustering. DESeq2 was executed on default settings to identify genes differentially expressed at host infection conditions relative to UG. We considered genes with an FDR-adjusted *P*-value < 0.05 and LFC ≥ 2 to be upregulated. Secretome genes upregulated at 4, 6, or 8 dpi were identified as our preliminary *Avr* effector candidates.

### Centromere inference and analysis

Centromere locations were determined based on strong inter-chromosomal interaction signals on Juicebox v2.17.00 Hi-C heatmap (Durand et al. 2016a) and confirmed by analyzing ONT-derived DNA methylation data (Supplemental Methods).

CDRs were located via visually selecting the largest hypomethylated region as this single pattern consistently appeared throughout all the inferred centromeres. Relative CDR position was calculated by dividing its midpoint coordinate by centromere length and then compared between haplotypes to detect CDR shifts. High-resolution alignment dotplots were generated in Gepard v2.1 to investigate the sequence composition at shifted CDRs (Krumsiek et al. 2007).

Centromeric TE enrichment was analyzed with permutation tests using our custom Python script. The rationale behind was to randomly reshuffle locations of target features (e.g., TEs) throughout a given chromosome or genome (5000 times) to remove their potential biological association with a region of interest (e.g., centromere), creating a null distribution of test statistics (e.g., TE coverage difference between centromere vs. noncentromere), which enabled a two-tailed hypothesis test for feature enrichment or depletion. *P*-values were defined as the proportion of permuted values equal to or more extreme than the observed value (<5% FDR).

### rDNA analysis

The canonical rDNA unit was defined by aligning reference ITS, 18S, and 5S of *Puccinia* species retrieved from databases including Gold Standard (Eenjes et al. 2022), EukRibo (Berney et al. 2022), and 5SrRNAdb (Szymanski et al. 2016) to distinguish conserved and variable elements. Long rRNA reads were aligned to confirm the transcribed units. Analyses of rDNA subtype variations and copy numbers were conducted on duplex and Illumina read alignments (Supplemental Methods).

The nuclear association of the two dominant rDNA subtypes was tested by analyzing rDNA Hi-C reads with a *k*-mer approach. Unique 31-mers of each subtype were identified using UniqueKMER (Chen et al. 2021). Subtype-specific 31-mers, along with their reverse complements, were used to tag rDNA Hi-C reads, whose paired Hi-C mates were fetched from the corresponding R1/R2 read file. Mates were mapped to the dikaryotic assembly with bwa-mem2 (Vasimuddin et al. 2019). Mapping locations and MAPQ scores were extracted with BEDTools *bamtobed* (Quinlan and Hall 2010) for plotting. This was repeated on subtype-nonspecific 31-mers as control. Note that if a read is tagged by both subtype-specific and unspecific *k*-mers, it is defined as subtype specific.

### Synteny and SV detection

Whole-genome alignment between haplotypes A and B was conducted with NUCmer (‐‐maxmatch -l 200 -b 500 -c 500). Only alignment blocks with >90% identity were retained. SyRI (Goel et al. 2019) was used to annotate SVs, syntenic and unaligned regions and then visualized with plotsr (Goel and Schneeberger 2022). To analyze features within and nearby SVs, BEDTools *slop* was used to extend SV coordinates by 2 kbp in both directions. Enrichment or depletion for genomic features, including TEs, genes, and subsets such as secretome and ASE-only genes, was statistically assessed through two-tailed permutation tests, as described above.

### Identification of hemizygous and heterozygous genes

Protein sequences of all annotated genes were analyzed using Proteinortho v6.3.1 (-synteny) (Lechner et al. 2011) to detect homologs between haplotypes. Genes lacking a hit on the alternative haplotype were considered hemizygous candidates. These were further filtered via reciprocal BLASTP (>70% identity; >70% query/subject coverage) to ensure the absence of alleles. One-to-one gene pairs with divergence values greater than zero (computed from codon-aware alignments generated using script “dN_dS_Pst134E.ipynb”) (Luo et al. 2024; https://github.com/ZhenyanLuo/codes-used-for-mating-type) were defined to be heterozygous biallelic.

### ASE analysis

We reformatted the Bambu gene-level read count matrices to test differential allele expression at heterozygous biallelic genes per condition using DESeq2 (Supplemental Methods). Allele pairs were grouped into different ASE status, as detailed in the Results. To assess nuclear dominance, allele read counts were normalized using DESeq2's median-of-ratio method and transformed into expression levels as log_10_(median of ratio + 1) and then averaged across replicates for haplotype comparisons. Overrepresentation of secretome genes in the ASE set (Diff2 and Diff4) relative to BUSCOs was evaluated via a two-sided Fisher's exact test on a 2 × 2 contingency table constructed per condition (<5% FDR). Methylation differences between ASE and non-ASE secretome alleles at the gene body and flanking regions were analyzed using a custom Python script (Supplemental Methods).

## Data access

All raw sequencing data generated in this study have been submitted to the NCBI BioProject database (https://www.ncbi.nlm.nih.gov/bioproject/) under accession number PRJNA1195871. All custom scripts for analyses and figures are available at GitHub (https://github.com/ritatam/Pst104EGenomeAnalysis) and as Supplemental Scripts. Genome assembly, rDNA sequences, and gene and TE annotations are available at Zenodo (https://doi.org/10.5281/zenodo.14885411) and as Supplemental Material.

## Supplemental Material

Supplement 1

Supplement 2

Supplement 3

Supplement 4

Supplement 5

Supplement 6

Supplement 7

Supplement 8

Supplement 9

Supplement 10

Supplement 11

Supplement 12

Supplement 13

Supplement 14

Supplement 15

Supplement 16

Supplement 17

Supplement 18
