# Supplementary material for: Long-read genomics reveal extensive nuclear-specific evolution and allele-specific expression in a dikaryotic fungus
Source: Genome Res. 2025 Jun;35(6):1364–76. doi: 10.1101/gr.280359.124 (PMC12129025; doi:10.1101/gr.280359.124)
Supplement: Supplement 1 [file Supplemental_Figures.pdf]

Supplemental Figures: Long-read genomics reveal extensive nuclear-specific evolution and allele-specific expression in a dikaryotic fungus

Rita Tam[1], Mareike Möller[1], Runpeng Luo[1], Zhenyan Luo[1], Ashley Jones[1], Sambasivam Periyannan[1,2,3], John P. Rathjen[1†], Benjamin Schwessinger[1†]

[1]Research School of Biology, Australian National University, Canberra ACT 2601, Australia

[2]Commonwealth Scientific and Industrial Research Organisation Agriculture and Food, Canberra ACT 2601, Australia

[3]School of Agriculture and Environmental Science, Centre for Crop Health, University of Southern Queensland, Toowoomba QLD 4350, Australia

Table of Contents

Supplemental Figure S1 2
Supplemental Figure S2 3
Supplemental Figure S3 4
Supplemental Figure S4 5
Supplemental Figure S5 6
Supplemental Figure S6 7
Supplemental Figure S7 8
Supplemental Figure S8 9
Supplemental Figure S9 10
Supplemental Figure S10 11
Supplemental Figure S11 12
Supplemental Figure S12 13
Supplemental Figure S13 14
Supplemental Figure S14 15
Supplemental Figure S15 16
Supplemental Figure S16 17

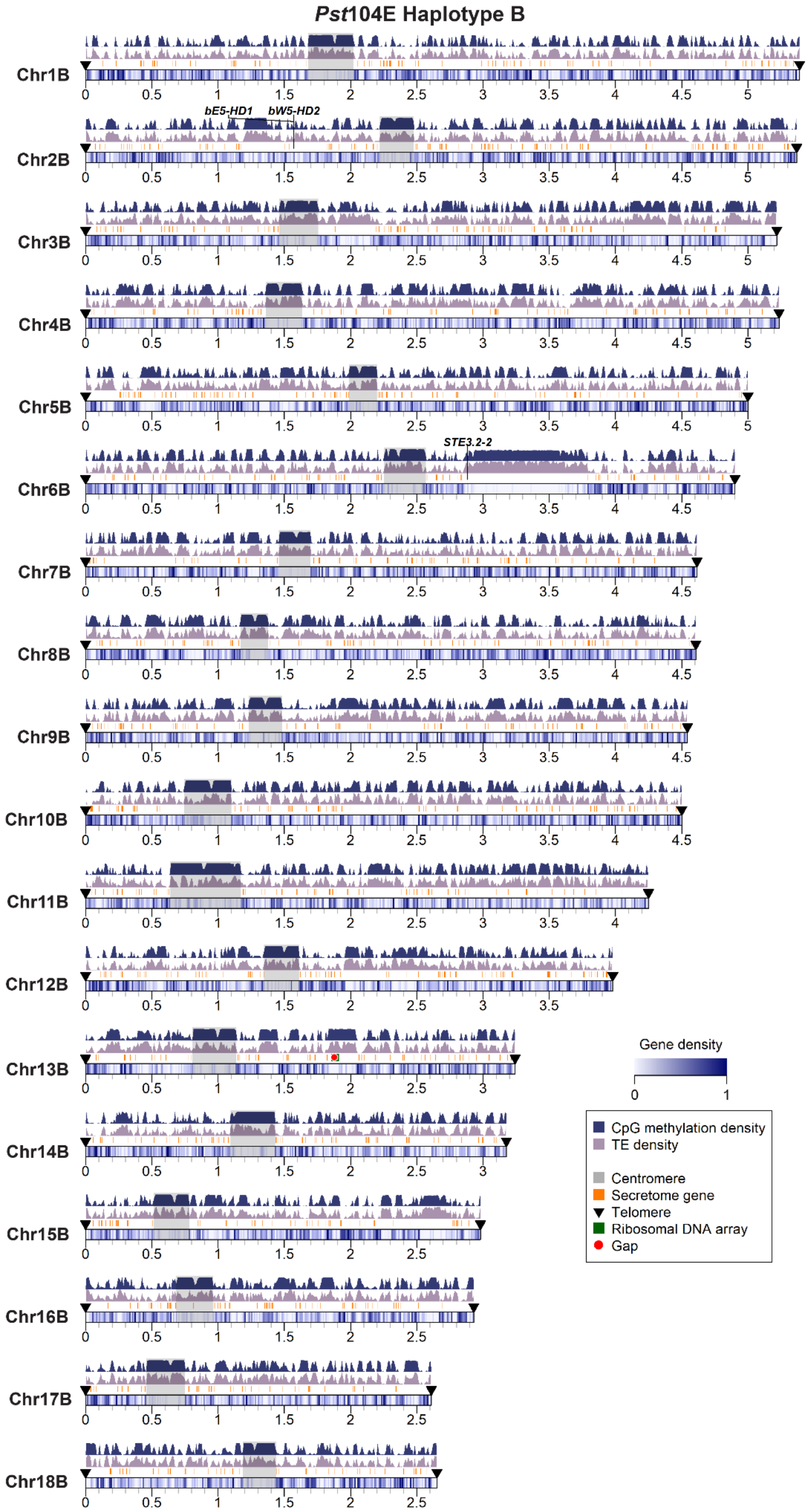

Supplemental Figure S1. Karyoplot of the 18 chromosomes of *Pst*104E haplotype B, showing density of CpG methylation and transposable elements (TEs) as peaks, and gene density as heatmaps within chromosome ideograms (10 kbp sliding windows). Locations of centromere, telomeres, secretome genes, ribosomal DNA array and assembly gaps are annotated as per legend.

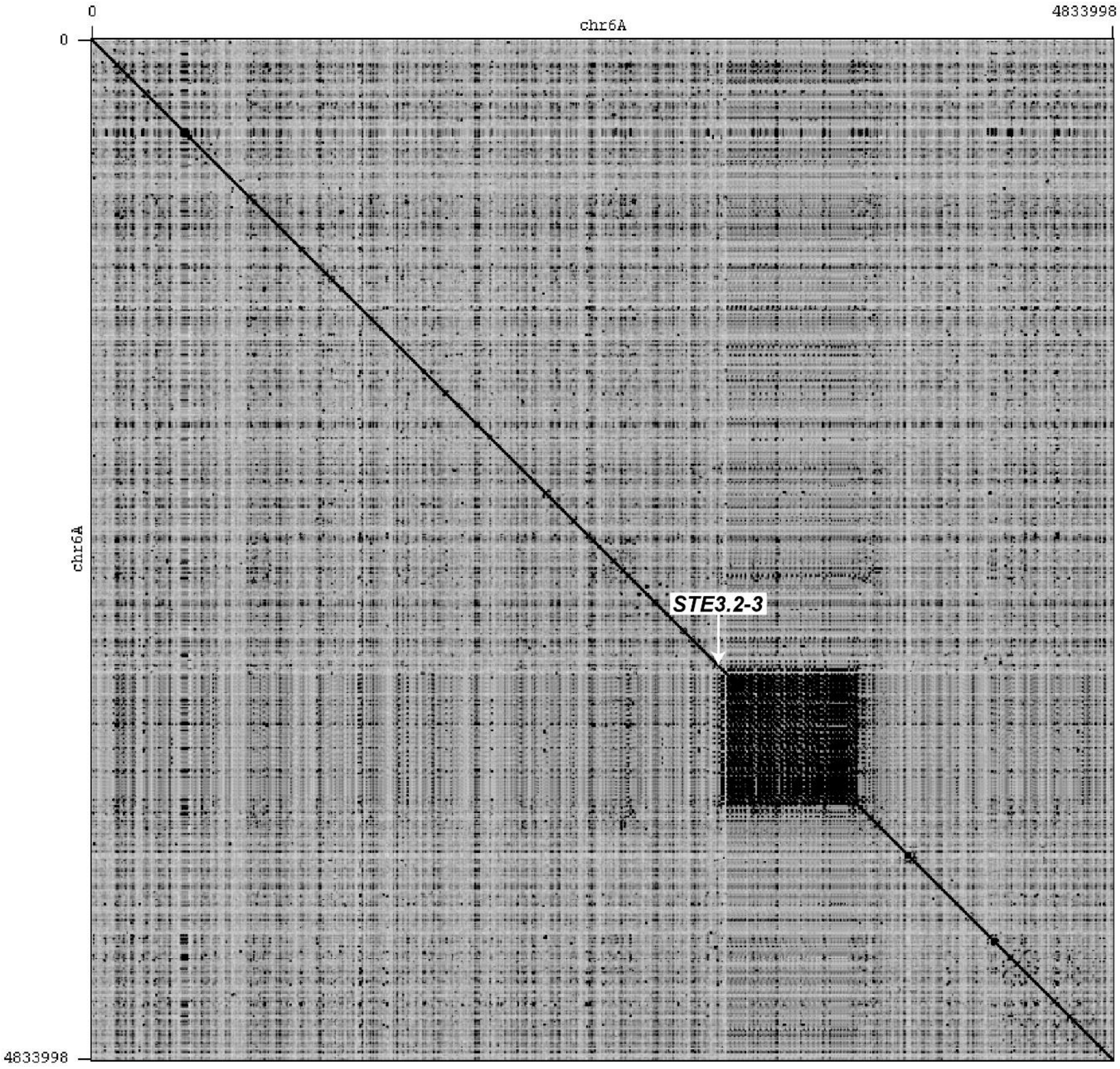

Supplemental Figure S2. Self-alignment dotplot of *Pst*104E Chromosome 6A. The large dark “patch” spanning ~650 kbp represents the highly repetitive LTR retrotransposon-rich region near the mating type *PR* locus, *STE3.2-3*, as previously documented (Luo et al. 2024). An assembly gap is present in this region potentially due to the high repetitiveness and near-identical repeat units. The corresponding region on haplotype B is completely assembled (not shown). Aligned and visualised in Gepard (Krumsiek et al. 2007).

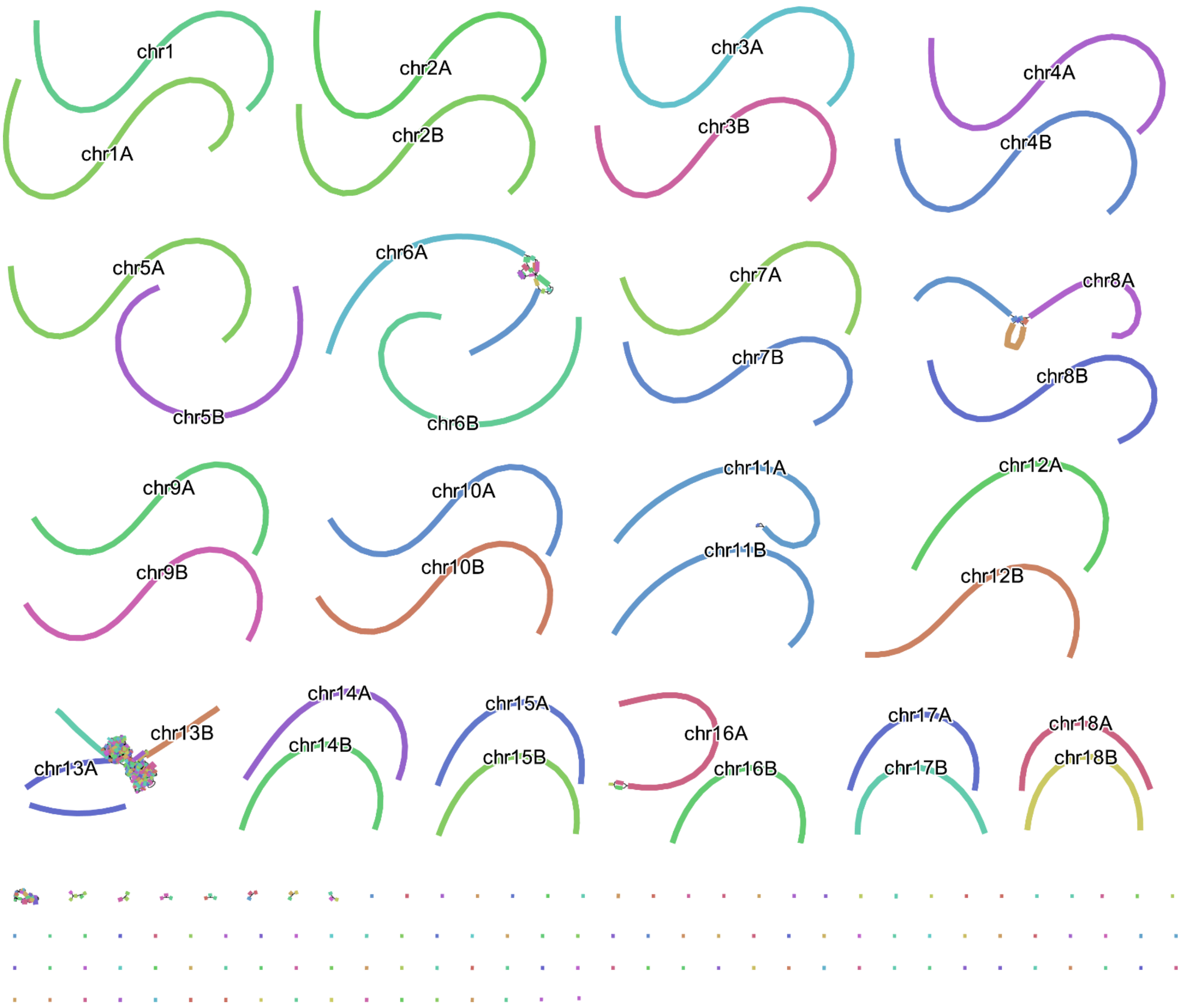

Supplemental Figure S3. Genome string graph of raw Verkko assembly of *Pst*104E. Most homologous chromosomes were directly resolved into two linear sequences. Tangles represent ambiguous connections at complex genomic regions, usually associated with long, highly identical repetitive sequences (e.g. rDNA arrays on Chr13). All identified assembly gaps coincided with these tangles (one gap on Chr6A, Chr13A and Chr13B; two gaps on Chr8A). The small unresolved graph bubble at the chromosomal end of Chr16A may indicate a large duplication event at the subtelomeric region leading to an unassembled telomere on its q-arm.

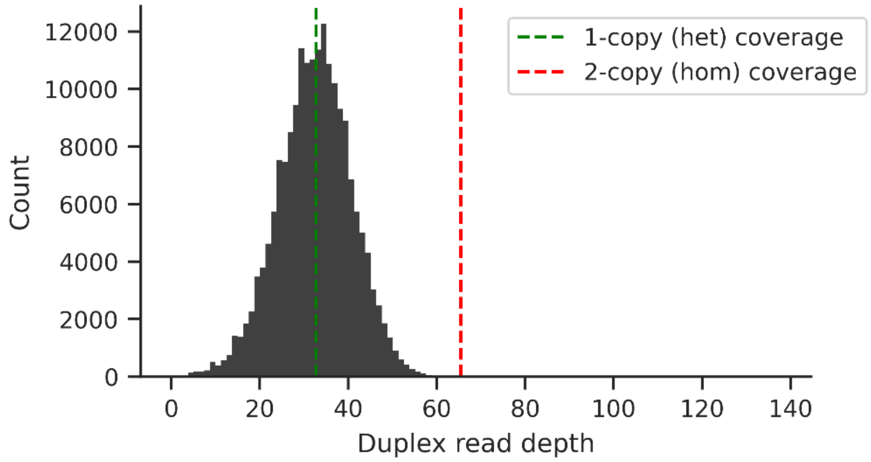

Supplemental Figure S4. Histogram of duplex read depth when mapped against the phased *Pst*104E assembly. If an assembly contains haplotype-collapsed regions, the reads derived from both haplotypes would map to the same location giving rise to 2x haploid coverage (red dotted line). Here, a single peak centres at the expected 1x haploid coverage (green dotted line), and no visible peak is present at the expected 2x haploid coverage, implying that the assembly is completely (or almost completely) phased.

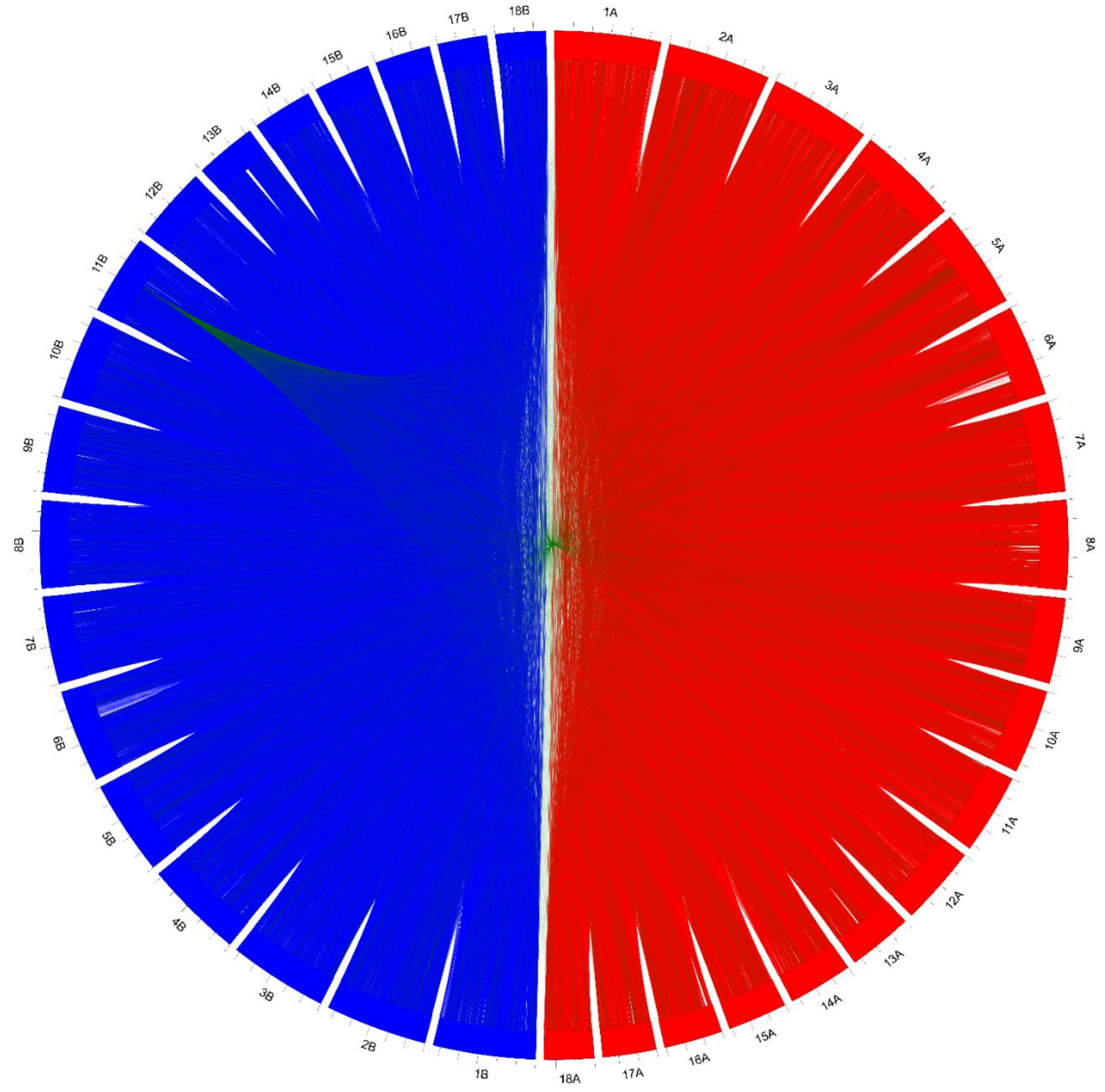

Supplemental Figure S5. Circos plot visualising within- and cross-haplotype Hi-C contact links for confirming phasing correctness of the *Pst*104E assembly. Each chromosome sequence was split into 20 kbp genomic windows, for which the rolling means of Hi-C links were computed. Hi-C links mapped within haplotype A are illustrated in red lines; haplotype B in blue lines. Cross-haplotype Hi-C links are illustrated in green lines. The 113th window on Chr11B shows frequent cross-haplotype links with haplotype A chromosomes implying a potential minor phase switch left in the assembly.

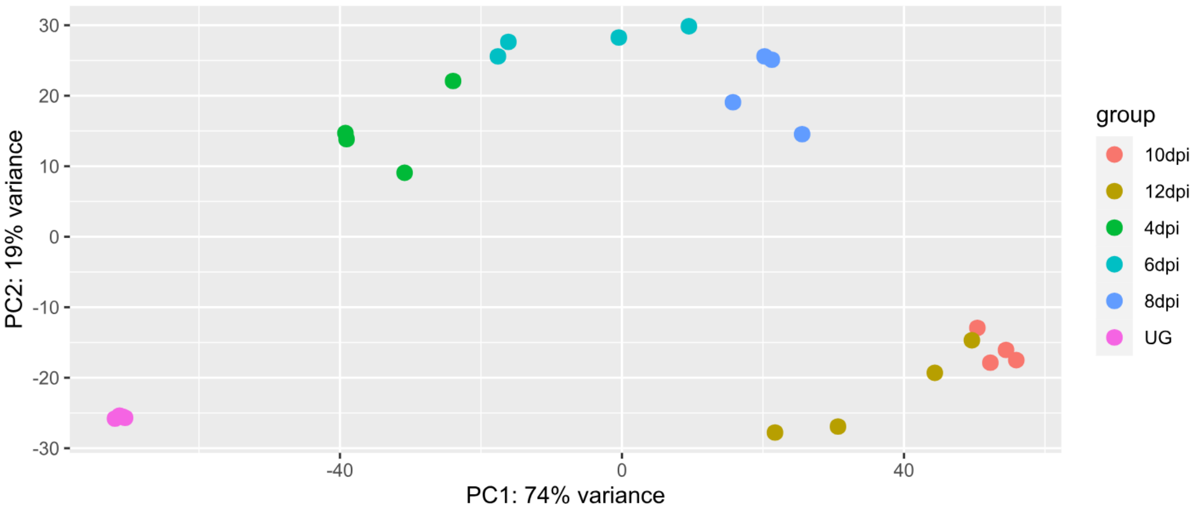

Supplemental Figure S6. Principle component analysis plot of the time-course ONT cDNA transcriptome dataset of *Pst*104E. Clear separations could be seen among the transcriptomic profiles derived from ungerminated (dormant) spores, early-mid host infection (4, 6 and 8 dpi) and late-infection (10 and 12 dpi) timepoints. UG: ungerminated urediniospores; dpi: days post infection.

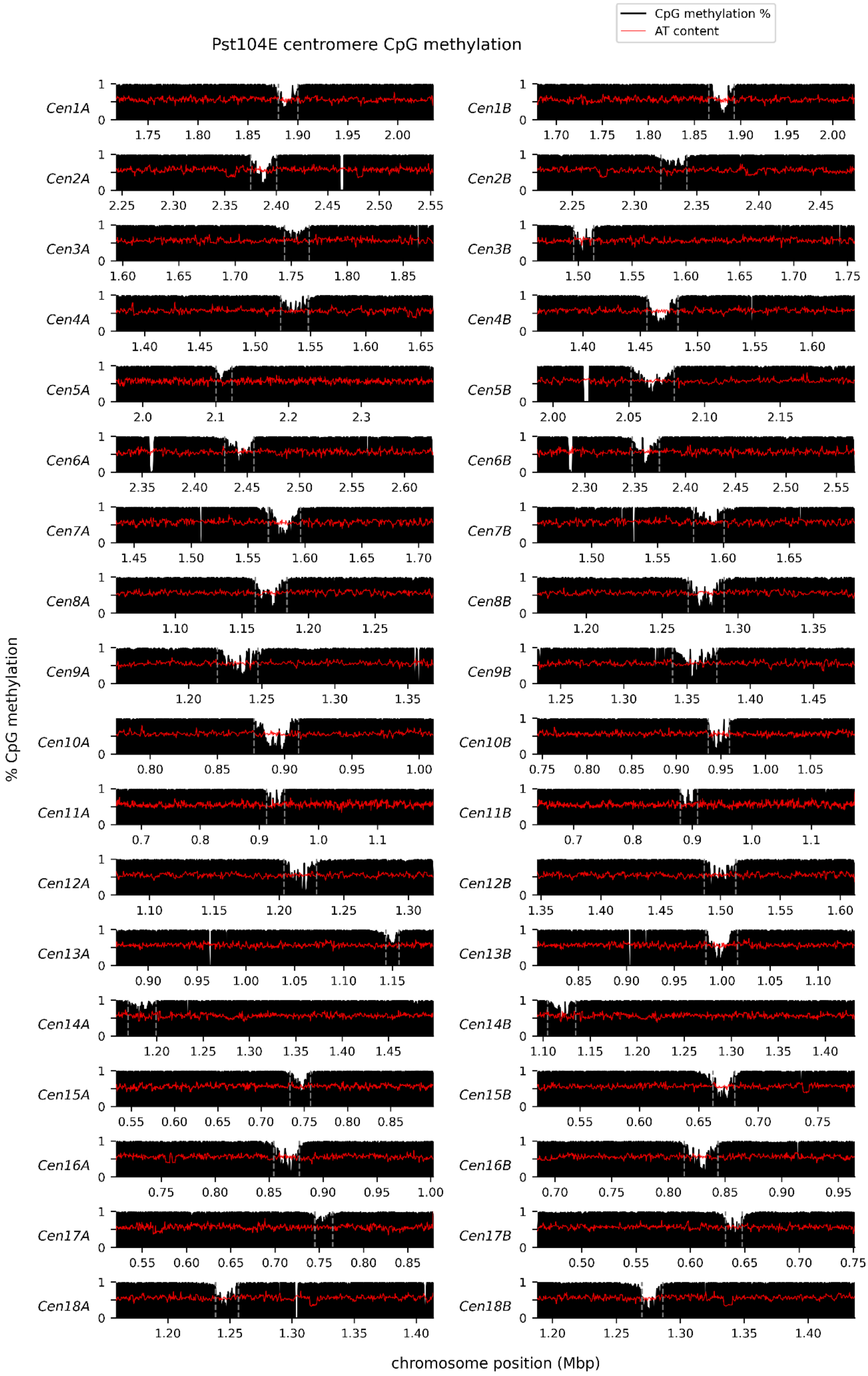

Supplemental Figure S7. CpG methylation profiles (black histogram) and % AT content (red line) of all 36 *Pst*104E centromeres. Rolling means were computed over 500 bp windows. A methylation depletion valley with the mean size of ~24.8 Kbp consistently appeared throughout all centromeres, indicating CDR signals (enclosed by grey dotted line).

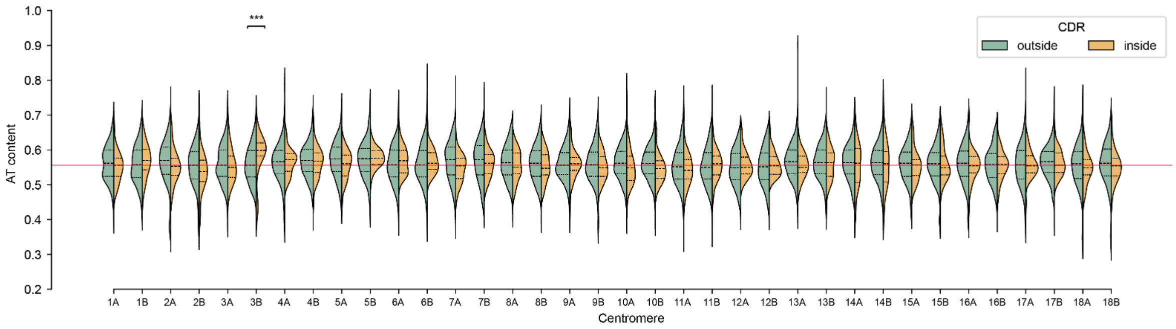

Supplemental Figure S8. Split violin plots comparing centromeric AT content in 500 bp windows inside (yellow) and outside (green) of centromere dip region (CDR), which is marked by the CpG methylation depletion signal in otherwise fully methylated centromere. The three dashed lines inside each violin denote the 25th, 50th (median) and 75th quartiles. Red solid line plotted at ~0.56 indicates the genome-wide AT content. This was analysed to check whether CDR signals were associated with elevated AT content. One-tailed Mann-Whitney U test (***, p<0.001).

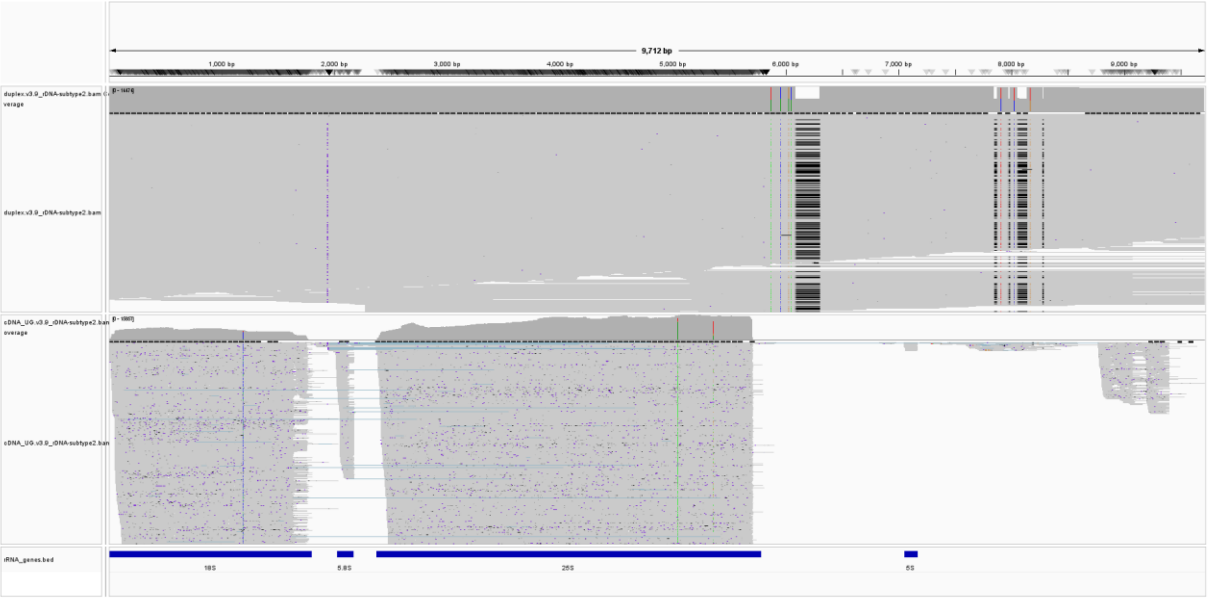

Supplemental Figure S9. IGV visualisation of ONT duplex (upper track) and cDNA (lower track) long-read alignments against the canonical rDNA repeat unit of *Pst*104E. Blue tracks at the bottom represent the annotated catalytic rRNA genes (18S, 5.8S, 25S and 5S) which were inferred from cDNA alignment for transcriptional activity, combined with results from homology search against rRNA gene references from a range of *Puccinia* species (see Methods). Duplex read alignment reveals two rDNA subtypes that harbour SNPs and length polymorphisms occurring at near-equal frequencies as reflected in the coverage track. The alternate bases revealed in the cDNA alignment did not line up with any high-confidence SNPs detected in aligned duplex reads. A possible explanation for these “misbasecall” profiles at these bases might be caused by rRNA modifications as described in (Begik et al. 2021).

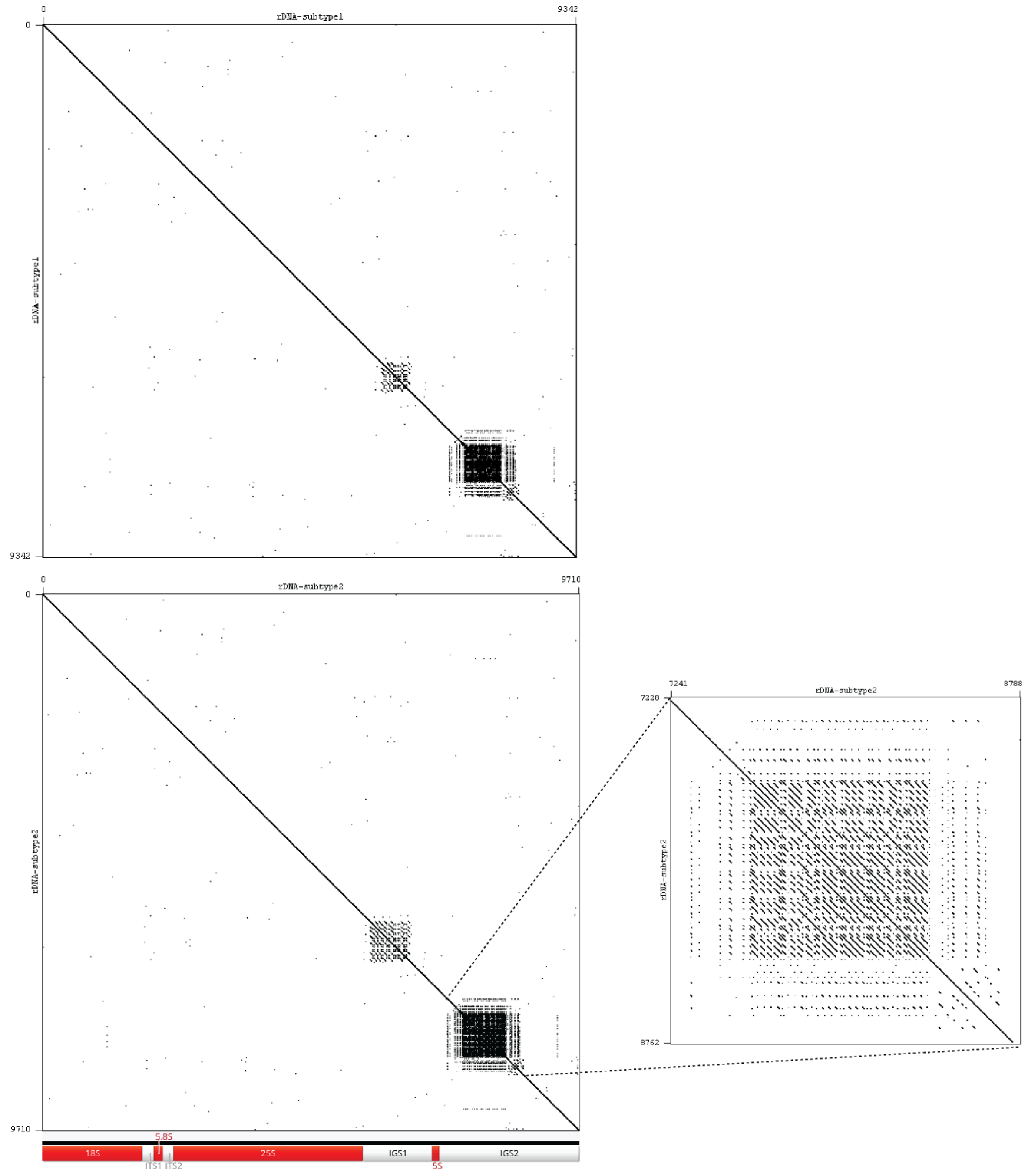

Supplemental Figure S10. Self-alignment dotplots of the two major rDNA subtypes (top: subtype #1, bottom: subtype #2) of *Pst*104E revealing two subrepeat regions nested within IGS1 and IGS2. Zoomed-in visualisation of the IGS2 subrepeats shows a microsatellite-like structure.

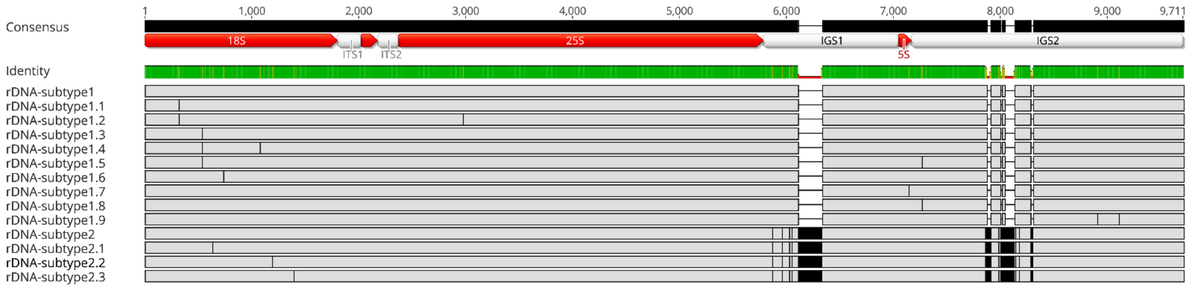

Supplemental Figure S11. Multiple sequence alignments of low-frequency variants of the dominant rDNA subtypes #1 and #2, numbered as #1.1–1.9 and #2.1–2.3. High-confidence SNPs were called from duplex read alignments against the canonical rDNA repeat using Bam-readcount (Khanna et al. 2022). SNP combinations were visually determined from aligned reads. Most SNPs accumulated at the 18S gene. Some subtype variants showed overlapping SNPs (e.g. #1.3–1.5) potentially implying spreading point mutations. See Supplemental Table S8 for SNP depths and the inferred copy numbers for each subtype.

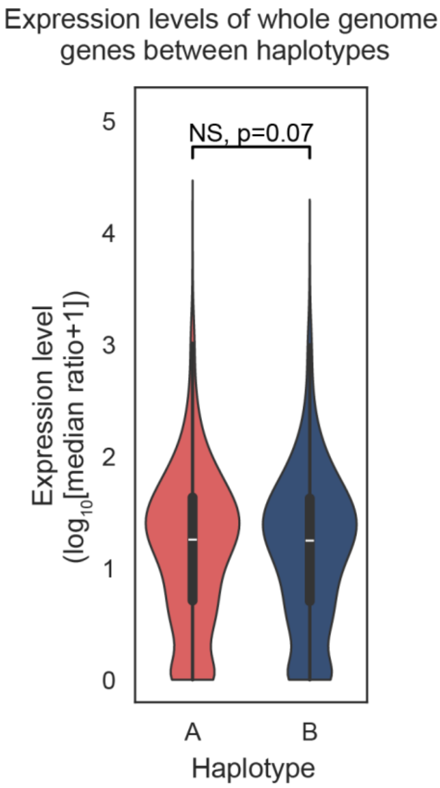

Supplemental Figure S12. Violin plots comparing expression level of genome-wide genes (not limited to paired alleles as opposed to Fig. 6B) belonging to the two nuclear haplotype A and B. Mann-Whitney U test (NS, non-significant).

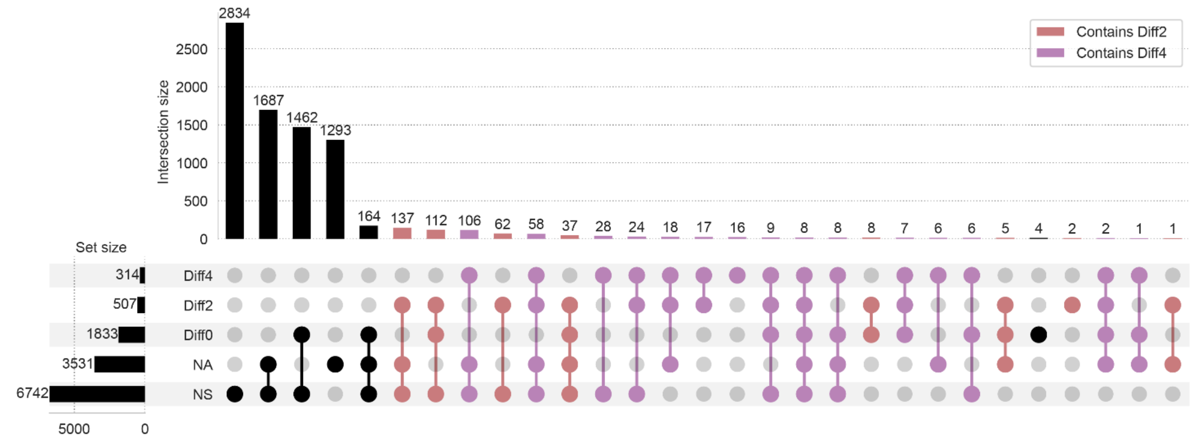

Supplemental Figure S13. Upset plot summarising different allele-specific expression (ASE) categories exhibited in all heterozygous biallelic gene pairs (n = 8,122) across the six sampling conditions. ASEs were categorised as followed: (1) no observed expression or no unambiguous transcript mapping at both alleles (NA); (2) no significant difference between alleles with false discovery rate (FDR) adjusted p-value > 0.05 (NS); and (3) significant difference between alleles with adjusted p-value < 0.05, indicating differential ASE. The differential ASE pairs were further classified based on $\log_2$ fold change (LFC): weak ASE, $|LFC|<2$ (Diff0); moderate ASE, $2 \leq |LFC| < 4$ (Diff2); and strong ASE, $|LFC|>4$ (Diff4). Highlighted intersections contain allele pairs that exhibited |LFC| greater than two (Diff2 and Diff4) in at least one condition, which were defined as ASE genes for the remainder of the analysis.

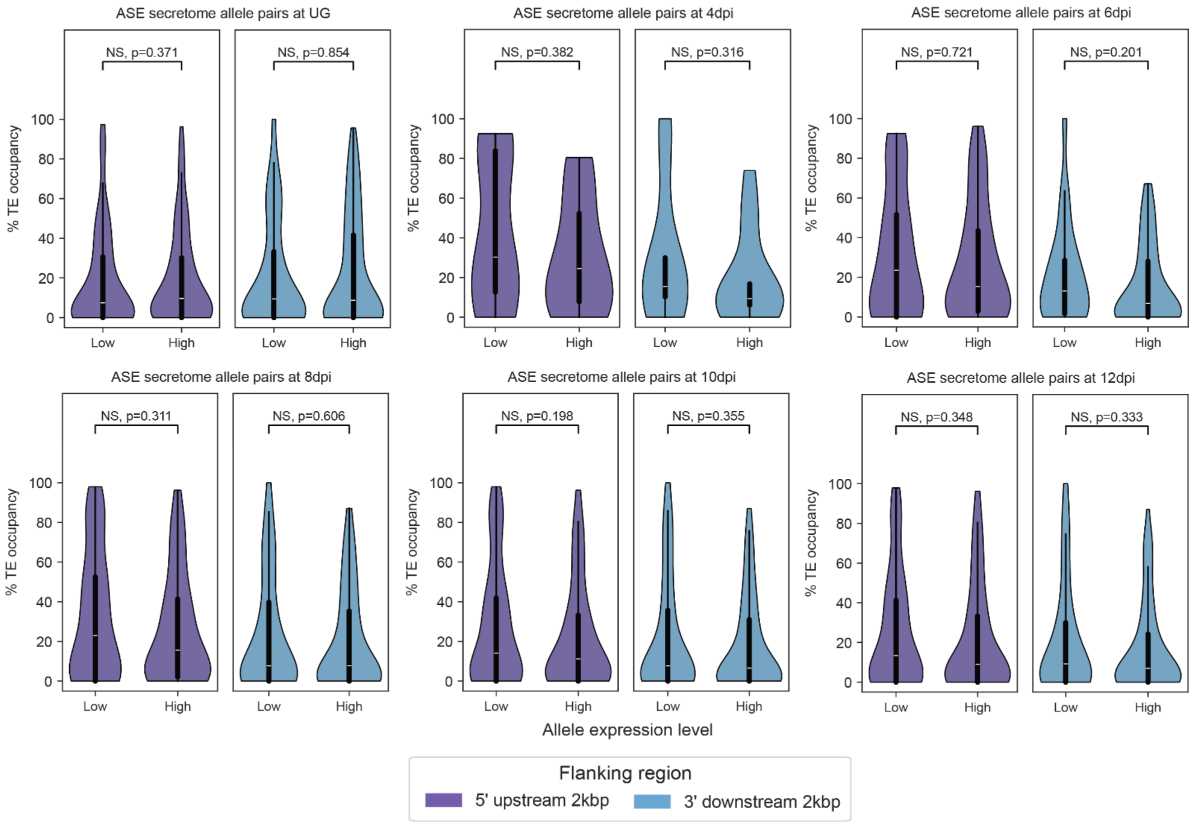

Supplemental Figure S14. Violin plots comparing the % TE occupancy between higher- and lower-expressed alleles of ASE secretome genes at their upstream and downstream 2 kbp flanking regions. Mann-Whitney U test (NS, non-significant). No difference in TE occupancy was detected between alleles.

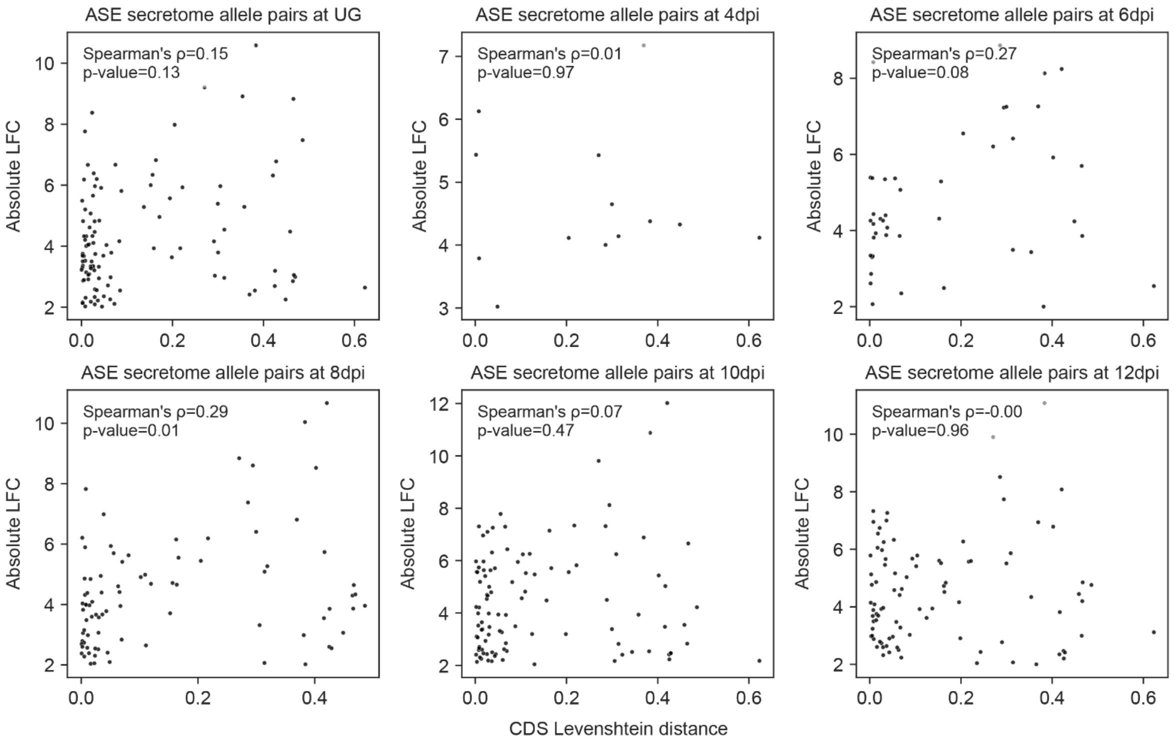

Supplemental Figure S15. Spearman's rho correlation analysis between CDS Levenshtein genetic distance and the expression |LFC| for secretome allele pairs displaying ASE at different conditions. No correlation was detected except for a weak positive correlation shown in the 8 dpi condition.

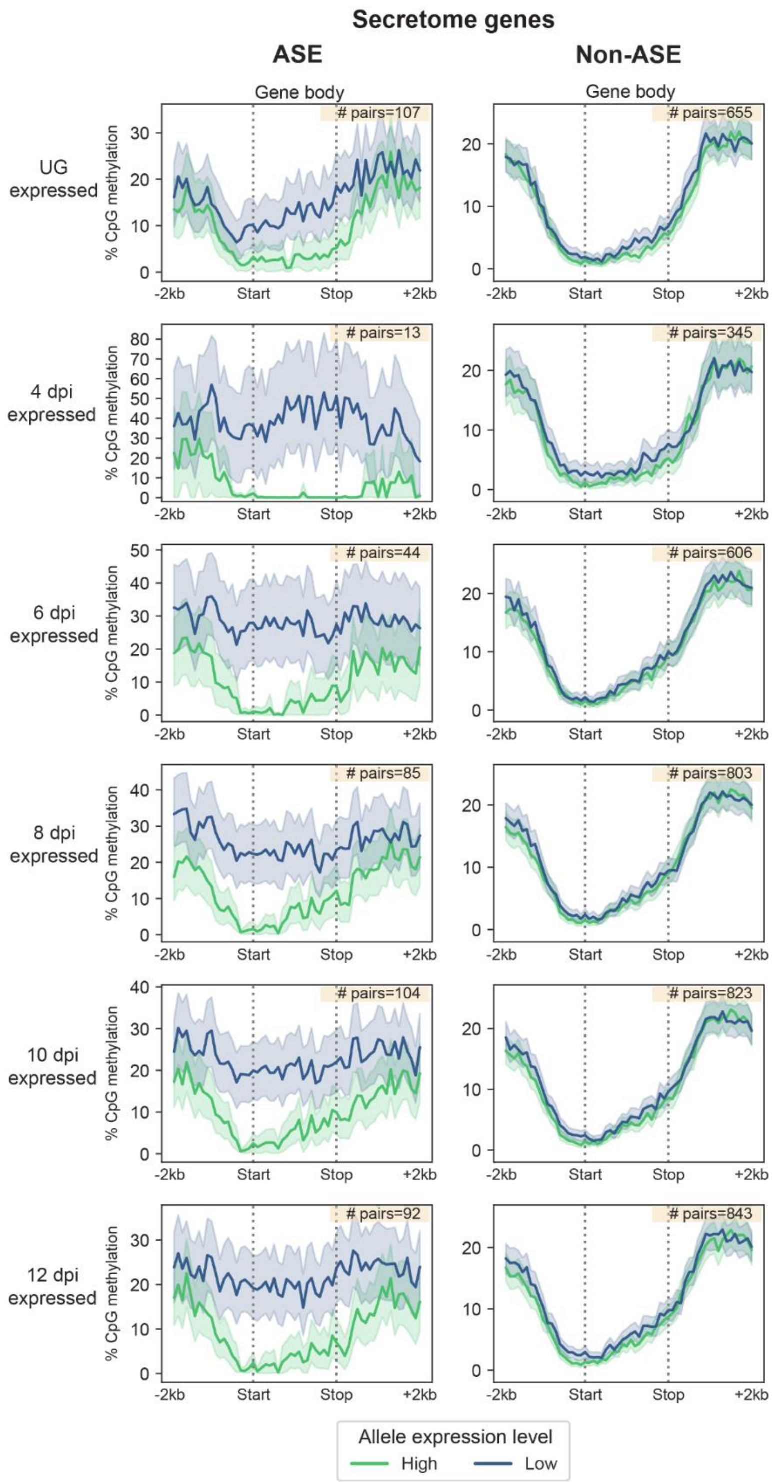

Supplemental Figure S16. Distribution of CpG methylation (sampled at UG) across ASE and non-ASE secretome gene bodies including +/- 2 kbp at each of the six sampling conditions. Gene body regions were defined as start and stop codon position, which were extended outwards by 2 kbp to include 5' upstream and 3' downstream flanking regions Each region was divided into 20 equally proportioned bins, totalling 60 bins per gene. The per-bin mean percentage coverage CpG methylation is plotted as solid line, with the 95% bootstrapping confidence interval as shaded area. The yellow inset indicates the number of heterozygous allele pairs included.
