# Supplementary material for: Long-read genomics reveal extensive nuclear-specific evolution and allele-specific expression in a dikaryotic fungus
Source: Genome Res. 2025 Jun;35(6):1364–76. doi: 10.1101/gr.280359.124 (PMC12129025; doi:10.1101/gr.280359.124)
Supplement: Supplement 2 [file Supplemental_Methods.pdf]

Supplemental Methods: Long-read genomics reveal extensive nuclear-specific evolution and allele-specific expression in a dikaryotic fungus

Rita Tam[1], Mareike Möller[1], Runpeng Luo[1], Zhenyan Luo[1], Ashley Jones[1], Sambasivam Periyannan[1,2,3], John P. Rathjen[1†], Benjamin Schwessinger[1†]

[1]Research School of Biology, Australian National University, Canberra ACT 2601, Australia

[2]Commonwealth Scientific and Industrial Research Organisation Agriculture and Food, Canberra ACT 2601, Australia

[3]School of Agriculture and Environmental Science, Centre for Crop Health, University of Southern Queensland, Toowoomba QLD 4350, Australia

Table of Contents

Supplemental Methods **2**
DNA extraction and long-read sequencing 2
Hi-C sequencing 2
Long-read cDNA library preparation and sequencing 2
Genome assembly and curation 3
TE annotation 4
Transcriptome assembly 4
Gene annotation 4
Centromere inference and analysis 5
Centromeric TE enrichment analysis 6
rDNA analysis 6
Synteny, structural rearrangements and variations detection 7
Identification of hemizygous and heterozygous genes 7
Allele-specific expression analysis 7
References **8**

Supplemental Methods

DNA extraction and long-read sequencing

High-molecular-weight genomic DNA was extracted from fresh Pst104E urediniospores as described previously (Schwessinger and Rathjen 2017) and size-selected for at 15kb using the BluePippin (Sage Science). Nanopore sequencing library was prepared using the Ligation Sequencing Kit v14 (SQK-LSK114), then sequenced on a PromethION sequencer, each library on a single R10.4.1 flowcell at 260 bps translocation speed. Sequencing was performed at the Biomolecular Resource Facility (BRF) at The Australian National University. Simplex reads were basecalled from raw signals with dorado v0.2.1 using the super accuracy (SUP) model “dna_r10.4.1_e8.2_260bps_sup@v4.1.0”. Duplex reads were paired with duplex-tools v0.3.1, then basecalled using the stereo model “dna_r10.4.1_e8.2_4khz_stereo@v1.1”. Seqkit v2.6.1 (Shen et al. 2016) was used to confirm the sequencing quality of each library’s output before concatenating them. NanoFilt v2.8.0 was used to trim and filter duplex reads (-q 30 -l 10000 --headcrop 75 --tailcrop 75), as well as simplex reads (-q 10 -l 40000). To address chimera, we performed all-versus-all duplex read alignment with minimap2 v2.26 (-x ava-ont) (Li 2018), followed by yacrd and fpa (Marijon et al. 2020) to conservatively split chimeric reads at zero coverage regions.

Hi-C sequencing

Spores were cross-linked with 1% formaldehyde and Hi-C library prepared with the Proximo Hi-C (Fungal) Kit KT6040 Protocol Version 4.0 (February 2021). Spore samples were cross-linked with 1% formaldehyde, quenched with 1% glycine, washed twice with 1x PBS and ground to fine powder using a Qiagen TissueLyser I. Tissue was sent to Phase Genomics (Seattle, WA, USA) for Hi-C library preparation (Proximo Hi-C (Fungal) Kit KT6040 Protocol Version 4.0 (February 2021), restriction enzymes used: DpnII, HinFI, MseI, DdeI) and sequencing. Sequencing (150 bp paired-end) was performed on an Illumina NovaSeq 6000 flowcell.

Long-read cDNA library preparation and sequencing

Infected leaves and urediniospores were ground to fine powder using a Qiagen TissueLyser II (25 Hz, 1 min). 1 mL of TRIzol (Invitrogen, 15596018) was added to each sample and RNA was extracted with the Zymo Research Direct-zol RNA Miniprep Plus Kit (Zymo Research, R2070), including DNase I treatment. RNA concentration was determined with the Qubit RNA BR Kit (Invitrogen, Q10211), RNA integrity and quality was assessed with the Qubit RNA IQ Assay Kit (Invitrogen, Q33221) and by agarose gel electrophoresis.

50 µg total RNA was used for poly-A enrichment with Dynabeads Oligo$(dT)_{25}$ (Invitrogen, 61005) according to manufacturer’s instructions for purifying mRNA from total RNA. First strand cDNA synthesis was based on a modified version of the ONT “Direct cDNA Sequencing V14 with SQK-LSK114” protocol. Second strand synthesis and RNA degradation were based on a modified NEB protocol (https://www.neb.com/en-au/protocols/2019/05/09/2nd-strand-cdna-synthesis-protocol-using-the-template-switching-rt-enzyme-mix). See Supplementary Notes for detailed descriptions. Barcoding and library preparation of samples were performed with the ONT Native Barcoding Kit 96 (SQK-NBD114.96) using approximately 100 ng cDNA as input. Barcoded samples were combined in different pools for ONT sequencing using three FLO-PRO114M flowcells. Sequencing was performed at the BRF at the Australian National University.

Genome assembly and curation

We used Verkko v1.3.1 (Rautiainen et al. 2023) to assemble 32x duplex (--hifi) and 117x simplex (--nano) reads following developers' recommendation for ONT-only assembly. To incorporate Hi-C, we ran the "gfase_wrapper.sh" script from an early version of Verkko's Hi-C phasing pipeline (https://github.com/Dmitry-Antipov/verkkohic), which employs bwa v0.7.17-r1188 (Li and Durbin 2009) to map Hi-C data against the raw assembly, and GFAse (Lorig-Roach et al. 2023) to perform haplotype phasing. The raw assembly consisted of 355 contigs and one gapped scaffold produced by Verkko totalling 168 Mbp. The raw assembly was aligned to the previously published *Pst*134E assembly (Schwessinger et al. 2022) using D-GENIES (Cabanettes and Klopp 2018) to identify chromosome-scale contigs and homologous pairs. Contaminant and mitochondrial contigs were identified using local BLAST v2.14.0 search against the NCBI's nt database (blastn -perc_identity 75 -evalue 1e-5) for removal. Contigs with mean window coverage lower than 5x were discarded.

Hi-C scaffolding was then performed using Juicer v2.0 (Durand et al. 2016) and 3D-DNA v180114 (Dudchenko et al. 2017), producing three additional chromosomal scaffolds (Chr6A, 8A and 13A). With the centromeres inferred from the Hi-C heatmaps, most chromosomes appeared to be acrocentric or submetacentric; we oriented them such that each begins from the shorter p-arm. We sorted the *Pst*104E chromosomes by the average length of each homologous pair starting from the longest to shortest.

We identified assembled telomeres by searching TTAGGG/CCCTAA motifs at the first and last 50bp of all scaffolds using "FindTelomeres.py" (https://github.com/JanaSperschneider/FindTelomeres) (Sperschneider 2024). Eight chromosomes had at least one telomere missing (chr2B, 5B, 6A, 8A, 10A, 11A, 13A and 16A), as seen in IGV with "show soft-clipped bases" option toggled on. To recover missing telomeres, we used the pre-release version of Teloclip (https://github.com/Adamtaranto/teloclip) (Taranto 2024) to extract UL reads aligned at contig ends and had soft-clipped overhangs harbouring at least two telomeric repeats, including the ~5 Kbp subtelomeric region for subsequent anchoring. These were locally assembled with Flye v2.9.1 (Kolmogorov et al. 2019), then stitched to the corresponding scaffold end. Only repeats supported by at least two reads were added. Reads were then mapped against the revised assembly to check if alignments correctly extended into the recovered ends.

For curation, we performed coverage check by separately mapping duplex and simplex reads back to the assembly with minimap2 (-ax map-ont) (Li 2018), then generated a genome-wide coverage plot via JVarkit's *wgscoverageplotter* (Lindenbaum 2015). For better resolution, we used bedtools' *makewindows* function (Quinlan and Hall 2010) to split contigs into 10kbp sliding windows with 2kbp overlapping interval, then computed per-base coverage averaged across each window with bamtocov (Birolo and Telatin 2022). A custom python script was used to pull out genomic coordinates of continuous windows with abnormally high or low coverage depth based on 5th and 95th percentile cutoffs, which we marked as discrepancies for inspection. These regions were marked as discrepancies for further investigation and manual correction where possible. Using this method, we detected and gap-filled a ~500 bp $(GAAAA)_n$ tandem repeat region on chr13B by locally assembling the UL reads. Finally, by aligning unplaced contigs against the chromosomal scaffolds, we discovered and manually corrected a ~10 kbp misassembly upstream of the chr13B rDNA array. After each curation step, we aligned duplex reads to the revised assembly to check if alignments agreed with the edits.

TE annotation

Each haplotype genome of *Pst*104E was annotated separately. TEs were predicted *de novo* using the REPET v3.0 pipeline, which consists of TEdenovo (Flutre et al. 2011) and TEannot (Quesneville et al. 2005). First, TEdenovo was run on default settings to detect repeats based on Repbase v27.06 (nt and aa databases), using the hidden Markov model profile bank from Pfam v35.0 (Finn et al. 2013) and Gypsy Database v2.0 (Lloréns et al. 2007) ("ProfilesBankForREPET_Pfam35.0_GypsyDB_2022.hmm"). Then, following REPET authors' recommendations for better annotation quality, two rounds of TEannot were performed. The first round was to define the total TE consensus library without feature detection and classification (steps 1, 2, 3 and 7). Consensuses that have at least two copies and one full-length copy (i.e. matching fragments aligned with >95% of the consensus length) were retained as validated TEs, which were then annotated in the second TEannot run (steps 1–5, 7 and 8). PASTEC (Hoede et al. 2014) classifications and statistics for each TE consensus were obtained from output files with the ".classif" and ".annotStatsPerTE.tab" extensions, respectively. Bedtools v.2.30.0 *maskfasta* (Quinlan and Hall 2010) was used to soft-mask the annotated TEs in the genome assembly.

Transcriptome assembly

Prior to gene annotation, we independently processed the previously published Illumina RNA-seq (Schwessinger et al. 2018) and our ONT long-read cDNA datasets to generate transcript evidence. Illumina RNA-seq was filtered and trimmed with fastp v0.23.4 (--detect_adapter_for_pe --cut_right --correction) (Chen et al. 2018). Processed reads were mapped to the dikaryotic assembly using HISAT2 v2.2.1 (--max-intronlen 3000 --dta) (Zhang et al. 2021). Aligned and paired reads (-F4 -f2) were partitioned into haplotype sets using samtools v1.18 (Li et al. 2009) and merged across replicates (n=3) per condition. Transcriptome assembly was then performed for each condition. For *Pst*104E, CYR32 and *Pst*87/66 samples, we used StringTie2 (v2.2.1) (Kovaka et al. 2019) to infer transcripts from splice alignments, with -s2 and -m50 applied to ensure capturing of shorter single-exon transcripts. Additionally for *Pst*104E, we assembled transcripts using reference-guided Trinity v2.9.1 (--genome_guided_max_intron 3000 --jaccard_clip) (Grabherr et al. 2011).

For the *Pst*104E ONT cDNA dataset, reads were trimmed with Porechop_ABI v0.5.0 (Bonenfant et al. 2023), then aligned to the dikaryotic assembly using minimap2 v2.26 in splice-aware mode (-ax splice -ub -G 3000 --secondary=no). Aligned reads were partitioned into haplotype sets and merged across replicates (n=4) per condition. To identify transcript structures from noisy long reads reference-guided and annotation-free, we employed two recently published tools: StringTie2 (-L -s2 -m50) for better single-exon transcript discovery, and ESPRESSO v1.4.0 (ESPRESSO_S.pl -Q0) (Gao et al. 2023) for improved splice site detection. All the transcript GTF annotations were merged across all *Pst* samples using StringTie2 (--merge). For *Pst*104E, we also extracted transcriptome FASTA sequences from the annotations using Gffread v0.12.7 (Pertea and Pertea 2020), and concatenated them to the Trinity assemblies.

Gene annotation

Gene annotation was carried out separately for each haplotype. Funannotate v1.8.15 (Jonathan and Jason 2023) was launched to train PASA on all preassembled *Pst*104E transcripts from ESPRESSO, StringTie2 and Trinity. CodingQuarry-PM (pathogen mode) v2.0 (Testa et al. 2015) was run on to predict genes from the merged transcript annotations. Both standard and dubious outputs were combined into a single CodingQuarry-PM gene set. Next, we executed funannotate *predict* (--optimize_augustus --ploidy 1 --repeats2evm) using inputs as followed: *Pst*104E transcriptome assemblies (--transcript_evidence); transcript

alignments (--rna_bam); transcript annotations from ESPRESSO and StringTie2 (--transcript_alignments), trained PASA (--pasa_gff), CodingQuarry-PM (--other_gff); as well as protein evidence from UniProtKB/Swiss-Prot (release 2023_04) (The UniProt Consortium 2023) and the previously published *Pst*104E proteome (Pst_104E_v13_ph_ctg.protein.fa) (Schwessinger et al. 2018). All evidence types were parsed to EvidenceModeler v1.1.1 (Haas et al. 2008) to produce weighted consensus gene structures. Evidence weights were configured as “augustus:4 hiq:6 genemark:1 pasa:10 codingquarry:0 snap:1 glimmerhmm:1 proteins:6 transcripts:6”, and “--other_gff:10” for CodingQuarry-PM input. Untranslated regions were inferred from long-read alignments using InGenAnnot *utr_refine*.

For functional annotation, we started by predicting secretome and effector genes using SignalP v6.0 (--organism eukarya --mode slow) (Teufel et al. 2022) and InGenAnnot v0.0.11 *effector_predictor* (Lapalu et al. 2023). InGenAnnot *rescue_effectors* was employed to find potential effector genes missed in the unannotated transcripts. To filter false positives, we used Phobius v1.01 (Käll et al. 2004) and TMHMM v2.0 (Krogh et al. 2001) to confirm the absence of transmembrane domain outside of the N-terminal signal peptide region; only hits predicted by both tools were removed as they are more likely to be biologically accurate. Seconary metabolite biosynthesis gene clusters were identified using antiSMASH v6.1.1 (Medema et al. 2011). InterProScan v5.64-96.0 (Blum et al. 2021; Jones et al. 2014) was run locally to predict protein functions using its defaulted member databases. All results were parsed to funannotate *annotate* to integrate functional annotations to the predicted genes. BUSCO completeness of the annotated genes was assessed in protein mode.

Centromere inference and analysis

Centromere locations were first estimated from the genome-wide Hi-C heatmap in Juicebox by identifying strong inter-chromosomal bowtie-like contact signals indicative of centromere-to-centromere interactions. To assess their methylation status, we called 5mCG modifications from a 400 bps ONT genomic dataset of *Pst*104E using dorado’s SUP model “dna_r10.4.1_e8.2_400bps_sup@v4.1.0”. Reads with methylation calls were mapped to the *Pst*104E assembly with minimap2 (-ax map-ont --secondary=no). Modkit v0.2.5 *pileup* (https://github.com/nanoporetech/modkit) was used to count modified CpGs in the reference using the aligned reads (--edge-filter 75 --combine-strands --bedgraph). This generates a bedGraph file that reports the fraction of reads exhibiting cytosine methylation at each reference CpG. We calculated mean methylation fractions over 500 bp windows and plotted them along each chromosome to confirm overlap between methylation peaks and the Hi-C bowtie signals, which we inferred as centromeres. To analyse their synteny, pairwise alignment between homologous centromeres was performed using MUMmer’s v4.0.0rc1 NUCmer tool (Marçais et al. 2018) with default settings. Alignment blocks longer than 100 bp with minimum sequence identity of 90% were retained and plotted in Dot (Sommer 2021).

We determined the locations and sizes of CDRs by visually selecting the largest hypomethylation region as this single pattern consistently appeared throughout all the centromeres. Relative CDR positions were calculated as the midpoint coordinate divided by centromere length, then compared between haplotypes to detect CDR shifts. To investigate sequence composition at shifted CDRs in greater detail, high-resolution alignment dotplots of homologous centromeres were generated using Gepard (Krumsiek et al. 2007). The associated TEs were inspected in Geneious and visualised as feature tracks using pyGenomeViz (Shimoyama 2024).

Centromeric TE enrichment analysis

Centromeric TE enrichment was analysed using a custom script employing permutation tests. The script takes in centromere and TE annotations, then applies bedtools *intersect* (-f 0.5) to identify TEs located in centromeric and non-centromeric regions along with their classifications. TE locations are then randomly shuffled along each chromosome in 5,000 permutations using bedtools *shuffle*. For every permutation, the coverage difference of a given TE superfamily between the centromeric and non-centromeric region is calculated. All permuted data generates a null distribution, enabling a two-tailed test for the statistical significance of centromeric enrichment or depletion per TE superfamily. P-values, defined as the proportion of permuted results equal to or more extreme than the observed, were adjusted for multiple testing using <5% FDR.

rDNA analysis

rDNA arrays were located using the whole-genome BLAST results as described above. We defined the canonical rDNA unit by integrating evidence from homology alignment and rRNA reads from the ONT cDNA library. Reference sequences of ITS, 18S and 5S regions from *Puccinia* species were retrieved from public databases including Gold Standard (Eenjes et al. 2022), EukRibo (Berney et al. 2022) and 5SrRNAdb (Szymanski et al. 2016), and aligned to the rDNA arrays to distinguish conserved and variable elements. Long rRNA reads were then used to refine rRNA gene boundaries. To suppress multimapping, all rDNA sequences were removed from the assembly, with a single rDNA repeat (starting from 18S) added as an extra "scaffold". All UG cDNA reads were mapped to the edited assembly with minimap2 in splice-aware mode, then visualised in IGV to reveal transcribed elements and confirm the canonical rDNA unit.

To capture rDNA sequence variations, duplex reads were aligned to the same edited assembly with minimap2, revealing two dominating subtypes. Reads were then mapped to these subtypes to identify low-frequency SNPs, which were called using bam-readcount v1.0.1 (Khanna et al. 2022) and parsed into a SNP information table with the included "parse_brc.py" script. SNPs were filtered with a 30x alternate base count threshold, as this reflects the lowest duplex read depth expected for single-copy subtypes based on the haploid genome coverage. SNPs detected at homopolymers were also excluded. SNP combinations were manually examined in read alignments, allowing us to reconstruct twelve low-frequency rDNA subtypes. Multiple sequence alignment among all subtypes was conducted using MAFFT v7.490 (Katoh et al. 2002; Katoh and Standley 2013). Inspired by (Sharma et al. 2022), the copy number of each rDNA subtype was estimated by normalising duplex and Illumina read depths (or SNP depths for low-frequency subtypes) to the mode per-base depth values of the whole genome, rather than the mean-based approach to minimise skewness.

The nuclear specificity of the two dominant rDNA subtypes was tested by analysing rDNA Hi-C reads with a k-mer approach. Unique 31-mers for each subtype were identified using UniqueKMER (Chen et al. 2020), with their reverse complements added via seqkit (Shen et al. 2016). Subtype-specific 31-mers were used to tag rDNA Hi-C reads with UNIX grep command. Using their read identifiers, paired Hi-C mates were fetched from the corresponding R1/R2 read file. Mates were mapped to the dikaryotic assembly with bwa-mem2 (Vasimuddin et al. 2019), and processed with bedtools *bamtobed* to extract mapping locations and MAPQ scores for plotting. This was repeated on subtype-unspecific 31-mers as control (note if a read is tagged by both subtype-specific and unspecific *k*-mers, it is defined as subtype-specific).

Synteny, structural rearrangements and variations detection

To assess interhaplotype synteny, we performed whole-genome alignment between haplotypes A (reference) and B (query) using NUCmer (--maxmatch -l 200 -b 500 -c 500). Alignment blocks with <90% identity were filtered out using MUMmer's delta-filter, and the resulting delta file was converted to alignment coordinates using show-coords (-THrd) for downstream SV calling. SyRI (Goel et al. 2019) was launched on default settings to annotate structural rearrangements (inversions, translocations and duplications), syntenic regions, and unaligned (sequences absent in one haplotype due to indels or excessive sequence divergence) regions. Visualisation of synteny and SVs between homologous chromosomes were generated by plotsr (Goel and Schneeberger 2022). To analyse features within and nearby SVs, bedtools *slop* was used to extend SV coordinates by 2 kbp in both directions. Enrichment or depletion for genomic features, including TEs, genes, and specific subsets like secretome-only and ASE-only genes, was statistically assessed through two-tailed permutation tests as described above.

Identification of hemizygous and heterozygous genes

Protein sequences from all annotated genes were analysed using Proteinortho v6.3.1 (Lechner et al. 2011) with the -synteny flag to detect homologs between haplotypes. Genes lacking a hit on the alternative haplotype were considered hemizygous candidates. These were further filtered via reciprocal BLASTp to ensure the absence of alleles. Candidates with a high-quality hit that had >70% identity and >70% query and subject coverage were omitted, producing the final hemizygous gene list, which we intersected with upregulated secretome genes for high-priority *Avr* candidates.

For heterozygous genes, we began by identifying one-to-one gene pairs from Proteinortho results. Using a script adapted from "dN_dS_Pst134E.ipynb" (https://github.com/ZhenyanLuo/codes-used-for-mating-type) (Luo et al. 2024), MUSCLE v3.8.31 (Edgar 2004) was run on default settings to perform codon-aware alignments between each gene pair. Synonymous (d_S) and non-synonymous (d_N) divergence values were then calculated using PAL2NAL v14 (Yang 2007), along with CDS and protein Levenshtein distances generated by editdistance v0.6.2. A gene pair was determined to be heterozygous biallelic if either its d_S or d_N value was greater than zero.

Allele-specific expression analysis

Having the paired allele information, we reformatted the bambu per-gene cDNA count matrices for testing condition-specific ASE. Each row represents an allele pair, while counts for one allele and the other across all samples appear as subsequent columns (48 in total). With haplotype A alleles set as the reference, differential expression between allele pairs was analysed for each condition using DESeq2 on default settings. The resulting |LFC| and adjusted p-values were used to categorise the allele pairs into different ASE status, as detailed in the Results. To assess nuclear dominance, allele read counts were normalised using DESeq2's median of ratio method and transformed into expression levels as $\log_{10}$(median of ratio+1). Mean allele expression levels were calculated by averaging across replicates for haplotype comparisons.

To test for the overrepresentation of secretome genes in ASE set (Diff2 and Diff4) compared to BUSCOs, six 2x2 contingency tables were constructed using counts of ASE or non-ASE secretome and BUSCO genes for each condition. Two-sided Fisher's exact tests of independence were performed using SciPy v1.9.3 to compute the odds ratio and p-values, corrected to <5% FDR.

CpG methylation differences between ASE and non-ASE secretome alleles were analysed using a custom python script. Gene body regions were defined based on the start and stop codon coordinates from CDS GFF3 annotations, then extended outwards by 2 kbp to include upstream and downstream flanking regions, generating three BED files per gene. Each region was divided into 20 equally proportioned bins, totaling 60 bins per gene. Mean methylation density was calculated per bin and averaged across higher- and lower-expression alleles for each condition. Bootstrapping with 1,000 replicates was used to compute 95% confidence intervals.

To investigate TE occupancy surrounding the ASE genes, overlapping TE fragments were merged with bedtools *merge*, followed by bedtools *intersect* (-wo) to report the number of bases covered by TEs within each 5' and 3' flanking region.

References

Begik O, Lucas MC, Pryszcz LP, Ramirez JM, Medina R, Milenkovic I, Cruciani S, Liu H, Vieira HGS, Sas-Chen A, et al. 2021. Quantitative profiling of pseudouridylation dynamics in native RNAs with nanopore sequencing. *Nat Biotechnol* **39**: 1278–1291.

Berney C, Henry N, Mahé F, Richter DJ, Vargas C de. 2022. EukRibo: a manually curated eukaryotic 18S rDNA reference database to facilitate identification of new diversity. 2022.11.03.515105. https://www.biorxiv.org/content/10.1101/2022.11.03.515105v1 (Accessed November 25, 2024).

Birolo G, Telatin A. 2022. BamToCov: an efficient toolkit for sequence coverage calculations. *Bioinformatics* **38**: 2617–2618.

Blum M, Chang H-Y, Chuguransky S, Grego T, Kandasaamy S, Mitchell A, Nuka G, Paysan-Lafosse T, Qureshi M, Raj S, et al. 2021. The InterPro protein families and domains database: 20 years on. *Nucleic Acids Res* **49**: D344–D354.

Bonenfant Q, Noé L, Touzet H. 2023. Porechop_ABI: discovering unknown adapters in Oxford Nanopore Technology sequencing reads for downstream trimming. *Bioinforma Adv* **3**: vbac085.

Cabanettes F, Klopp C. 2018. D-GENIES: dot plot large genomes in an interactive, efficient and simple way. *PeerJ* **6**: e4958.

Chen S, He C, Li Y, Li Z, Melançon CE. 2020. *A Computational Toolset for Rapid Identification of SARS-CoV-2, other Viruses, and Microorganisms from Sequencing Data*. Bioinformatics http://biorxiv.org/lookup/doi/10.1101/2020.05.12.092163 (Accessed October 9, 2023).

Chen S, Zhou Y, Chen Y, Gu J. 2018. fastp: an ultra-fast all-in-one FASTQ preprocessor. *Bioinformatics* **34**: i884–i890.

Dudchenko O, Batra SS, Omer AD, Nyquist SK, Hoeger M, Durand NC, Shamim MS, Machol I, Lander ES, Aiden AP, et al. 2017. De novo assembly of the Aedes aegypti genome using Hi-C yields chromosome-length scaffolds. *Science* **356**: 92–95.

Durand NC, Shamim MS, Machol I, Rao SSP, Huntley MH, Lander ES, Aiden EL. 2016. Juicer Provides a One-Click System for Analyzing Loop-Resolution Hi-C Experiments. *Cell Syst* **3**: 95–98.

Edgar RC. 2004. MUSCLE: multiple sequence alignment with high accuracy and high throughput. *Nucleic Acids Res* **32**: 1792–1797.

Eenjes T, Hu Y, Irinyi L, Hoang MTV, Smith LM, Linde CC, Milgate AW, Meyer W, Stone EA, Rathjen JP, et al. 2022. Linked machine learning classifiers improve species classification of fungi when using error-prone long-reads on extended metabarcodes. 2021.05.01.442223. https://www.biorxiv.org/content/10.1101/2021.05.01.442223v4 (Accessed November 25, 2024).

Finn RD, Bateman A, Clements J, Coggill P, Eberhardt RY, Eddy SR, Heger A, Hetherington K, Holm L, Mistry J, et al. 2013. Pfam: the protein families database. *Nucleic Acids Res* **42**: D222.

Flutre T, Duprat E, Feuillet C, Quesneville H. 2011. Considering Transposable Element Diversification in De Novo Annotation Approaches ed. Y. Xu. *PLoS ONE* **6**: e16526.

Gao Y, Wang F, Wang R, Kutschera E, Xu Y, Xie S, Wang Y, Kadash-Edmondson KE, Lin L, Xing Y. 2023. ESPRESSO: Robust discovery and quantification of transcript isoforms from error-prone long-read RNA-seq data. *Sci Adv* **9**: eabq5072.

Goel M, Schneeberger K. 2022. plotsr: visualizing structural similarities and rearrangements between multiple genomes. *Bioinformatics* **38**: 2922–2926.

Goel M, Sun H, Jiao W-B, Schneeberger K. 2019. SyRI: finding genomic rearrangements and local sequence differences from whole-genome assemblies. *Genome Biol* **20**: 277.

Grabherr MG, Haas BJ, Yassour M, Levin JZ, Thompson DA, Amit I, Adiconis X, Fan L, Raychowdhury R, Zeng Q, et al. 2011. Trinity: reconstructing a full-length transcriptome without a genome from RNA-Seq data. *Nat Biotechnol* **29**: 644.

Haas BJ, Salzberg SL, Zhu W, Pertea M, Allen JE, Orvis J, White O, Buell CR, Wortman JR. 2008. Automated eukaryotic gene structure annotation using EVidenceModeler and the Program to Assemble Spliced Alignments. *Genome Biol* **9**: R7.

Hoede C, Arnoux S, Moisset M, Chaumier T, Inizan O, Jamilloux V, Quesneville H. 2014. PASTEC: An Automatic Transposable Element Classification Tool ed. R. Cordaux. *PLoS ONE* **9**: e91929.

Jones P, Binns D, Chang H-Y, Fraser M, Li W, McAnulla C, McWilliam H, Maslen J, Mitchell A, Nuka G, et al. 2014. InterProScan 5: genome-scale protein function classification. *Bioinformatics* **30**: 1236–1240.

Käll L, Krogh A, Sonnhammer ELL. 2004. A combined transmembrane topology and signal peptide prediction method. *J Mol Biol* **338**: 1027–1036.

Katoh K, Misawa K, Kuma K, Miyata T. 2002. MAFFT: a novel method for rapid multiple sequence alignment based on fast Fourier transform. *Nucleic Acids Res* **30**: 3059–3066.

Katoh K, Standley DM. 2013. MAFFT Multiple Sequence Alignment Software Version 7: Improvements in Performance and Usability. *Mol Biol Evol* **30**: 772–780.

Khanna A, Larson DE, Srivatsan SN, Mosior M, Abbott TE, Kiwala S, Ley TJ, Duncavage EJ, Walter MJ, Walker JR, et al. 2022. Bam-readcount - rapid generation of basepair-resolution sequence metrics. *J Open Source Softw* **7**: 3722.

Kolmogorov M, Yuan J, Lin Y, Pevzner PA. 2019. Assembly of long, error-prone reads using repeat graphs. *Nat Biotechnol* **37**: 540–546.

Kovaka S, Zimin AV, Pertea GM, Razaghi R, Salzberg SL, Pertea M. 2019. Transcriptome assembly from long-read RNA-seq alignments with StringTie2. *Genome Biol* **20**: 278.

Krogh A, Larsson B, von Heijne G, Sonnhammer ELL. 2001. Predicting transmembrane protein topology with a hidden markov model: application to complete genomes1. *J Mol Biol* **305**: 567–580.

Krumsiek J, Arnold R, Rattei T. 2007. Gepard: a rapid and sensitive tool for creating dotplots on genome scale. *Bioinformatics* **23**: 1026–1028.

Lapalu N, Lamothe L, Petit Y, Genissel A, Delude C, Feurtey A, Abraham LN, Smith D, King R, Renwick A, et al. 2023. *Improved gene annotation of the fungal wheat pathogen* Zymoseptoria tritici *based on combined Iso-Seq and RNA-Seq evidence*. Genomics http://biorxiv.org/lookup/doi/10.1101/2023.04.26.537486 (Accessed June 5, 2023).

Lechner M, Findeiß S, Steiner L, Marz M, Stadler PF, Prohaska SJ. 2011. Proteinortho: Detection of (Co-)orthologs in large-scale analysis. *BMC Bioinformatics* **12**: 124.

Li H. 2018. Minimap2: pairwise alignment for nucleotide sequences. *Bioinformatics* **34**: 3094–3100.

Li H, Durbin R. 2009. Fast and accurate short read alignment with Burrows–Wheeler transform. *Bioinformatics* **25**: 1754.

Li H, Handsaker B, Wysoker A, Fennell T, Ruan J, Homer N, Marth G, Abecasis G, Durbin R, 1000 Genome Project Data Processing Subgroup. 2009. The Sequence Alignment/Map format and SAMtools. *Bioinformatics* **25**: 2078–2079.

Lindenbaum P. 2015. JVarkit: java-based utilities for Bioinformatics. https://figshare.com/articles/journal_contribution/JVarkit_java_based_utilities_for_Bioinformatics/1425030/1 (Accessed November 20, 2024).

Lloréns C, Futami R, Bezemer D, Moya A. 2007. The Gypsy Database (GyDB) of mobile genetic elements. *Nucleic Acids Res* **36**: D38.

Lorig-Roach R, Meredith M, Monlong J, Jain M, Olsen H, McNulty B, Porubsky D, Montague T, Lucas J, Condon C, et al. 2023. *Phased nanopore assembly with Shasta and modular graph phasing with GFAse*. Bioinformatics http://biorxiv.org/lookup/doi/10.1101/2023.02.21.529152 (Accessed April 2, 2023).

Luo Z, McTaggart A, Schwessinger B. 2024. Genome biology and evolution of mating-type loci in four cereal rust fungi ed. T. Giraud. *PLOS Genet* **20**: e1011207.

Marçais G, Delcher AL, Phillippy AM, Coston R, Salzberg SL, Zimin A. 2018. MUMmer4: A fast and versatile genome alignment system. *PLoS Comput Biol* **14**: e1005944.

Marijon P, Chikhi R, Varré J-S. 2020. yacrd and fpa: upstream tools for long-read genome assembly. *Bioinforma Oxf Engl* **36**: 3894–3896.

Medema MH, Blin K, Cimermancic P, Jager V de, Zakrzewski P, Fischbach MA, Weber T, Takano E, Breitling R. 2011. antiSMASH: rapid identification, annotation and analysis of secondary metabolite biosynthesis gene clusters in bacterial and fungal genome sequences. *Nucleic Acids Res* **39**: W339.

Pertea G, Pertea M. 2020. GFF Utilities: GffRead and GffCompare. https://f1000research.com/articles/9-304 (Accessed November 14, 2024).

Quesneville H, Bergman CM, Andrieu O, Autard D, Nouaud D, Ashburner M, Anxolabehere D. 2005. Combined Evidence Annotation of Transposable Elements in Genome Sequences. *PLoS Comput Biol* **1**: e22.

Quinlan AR, Hall IM. 2010. BEDTools: a flexible suite of utilities for comparing genomic features. *Bioinformatics* **26**: 841–842.

Rautiainen M, Nurk S, Walenz BP, Logsdon GA, Porubsky D, Rhie A, Eichler EE, Phillippy AM, Koren S. 2023. Telomere-to-telomere assembly of diploid chromosomes with Verkko. *Nat Biotechnol* 1–9.

Schwessinger B, Jones A, Albekaa M, Hu Y, Mackenzie A, Tam R, Nagar R, Milgate A, Rathjen JP, Periyannan S. 2022. A Chromosome Scale Assembly of an Australian Puccinia striiformis f. sp. tritici Isolate of the PstS1 Lineage. *Mol Plant-Microbe Interactions®* **35**: 293–296.

Schwessinger B, Rathjen JP. 2017. Extraction of High Molecular Weight DNA from Fungal Rust Spores for Long Read Sequencing. In *Wheat Rust Diseases: Methods and Protocols* (ed. S. Periyannan), pp. 49–57, Springer, New York, NY https://doi.org/10.1007/978-1-4939-7249-4_5 (Accessed December 2, 2024).

Schwessinger B, Sperschneider J, Cuddy WS, Garnica DP, Miller ME, Taylor JM, Dodds PN, Figueroa M, Park RF, Rathjen JP. 2018. A Near-Complete Haplotype-Phased Genome of the Dikaryotic Wheat Stripe Rust Fungus *Puccinia striiformis* f. sp. *tritici* Reveals High Interhaplotype Diversity ed. A. Di Pietro. *mBio* **9**: e02275-17.

Sharma D, Denmat SH-L, Matzke NJ, Hannan K, Hannan RD, O’Sullivan JM, Ganley ARD. 2022. A new method for determining ribosomal DNA copy number shows differences between Saccharomyces cerevisiae populations. *Genomics* **114**: 110430.

Shen W, Le S, Li Y, Hu F. 2016. SeqKit: A Cross-Platform and Ultrafast Toolkit for FASTA/Q File Manipulation. *PLOS ONE* **11**: e0163962.

Shimoyama Y. 2024. pyGenomeViz: A genome visualization python package for comparative genomics. https://github.com/moshi4/pyGenomeViz (Accessed November 25, 2024).

Sommer D. 2021. dsommer/dot. https://github.com/dsommer/dot (Accessed November 11, 2024).

Sperschneider J. 2024. JanaSperschneider/FindTelomeres. https://github.com/JanaSperschneider/FindTelomeres (Accessed November 13, 2024).

Szymanski M, Zielezinski A, Barciszewski J, Erdmann VA, Karlowski WM. 2016. 5SRNAdb: an information resource for 5S ribosomal RNAs. *Nucleic Acids Res* **44**: D180–D183.

Taranto A. 2024. Adamtaranto/teloclip. https://github.com/Adamtaranto/teloclip (Accessed November 25, 2024).

Testa AC, Hane JK, Ellwood SR, Oliver RP. 2015. CodingQuarry: highly accurate hidden Markov model gene prediction in fungal genomes using RNA-seq transcripts. *BMC Genomics* **16**: 170.

Teufel F, Almagro Armenteros JJ, Johansen AR, Gíslason MH, Pihl SI, Tsirigos KD, Winther O, Brunak S, Von Heijne G, Nielsen H. 2022. SignalP 6.0 predicts all five types of signal peptides using protein language models. *Nat Biotechnol* **40**: 1023–1025.

The UniProt Consortium. 2023. UniProt: the Universal Protein Knowledgebase in 2023. *Nucleic Acids Res* **51**: D523–D531.

Vasimuddin Md, Misra S, Li H, Aluru S. 2019. Efficient Architecture-Aware Acceleration of BWA-MEM for Multicore Systems. In *2019 IEEE International Parallel and Distributed Processing Symposium (IPDPS)*, pp. 314–324 https://ieeexplore.ieee.org/document/8820962 (Accessed November 25, 2024).

Yang Z. 2007. PAML 4: phylogenetic analysis by maximum likelihood. *Mol Biol Evol* **24**: 1586–1591.

Zhang Y, Park C, Bennett C, Thornton M, Kim D. 2021. Rapid and accurate alignment of nucleotide conversion sequencing reads with HISAT-3N. *Genome Res* **31**: 1290–1295.

codes-used-for-mating-type/4. Investigation of recombination suppression in MAT loci at main · ZhenyanLuo/codes-used-for-mating-type. https://github.com/ZhenyanLuo/codes-used-for-mating-type/tree/main/4.%20Investigation%20of%20recombination%20suppression%20in%20MAT%20loci (Accessed September 19, 2024a).

nextgenusfs/funannotate: Eukaryotic Genome Annotation Pipeline. https://github.com/nextgenusfs/funannotate (Accessed June 11, 2024b).
