# Supplementary material for: Long-read genomics reveal extensive nuclear-specific evolution and allele-specific expression in a dikaryotic fungus
Source: Genome Res. 2025 Jun;35(6):1364–76. doi: 10.1101/gr.280359.124 (PMC12129025; doi:10.1101/gr.280359.124)
Supplement: Supplement 3 [file Supplemental_Note.pdf]

Supplemental Note: Long-read genomics reveal extensive nuclear-specific evolution and allele-specific expression in a dikaryotic fungus

Rita Tam[1], Mareike Möller[1], Runpeng Luo[1], Zhenyan Luo[1], Ashley Jones[1], Sambasivam Periyannan[1,2,3], John P. Rathjen[1†], Benjamin Schwessinger[1†]

[1]Research School of Biology, Australian National University, Canberra ACT 2601, Australia

[2]Commonwealth Scientific and Industrial Research Organisation Agriculture and Food, Canberra ACT 2601, Australia

[3]School of Agriculture and Environmental Science, Centre for Crop Health, University of Southern Queensland, Toowoomba QLD 4350, Australia

Supplemental Note

Investigation of association between secretome gene ASE and genetic factors

We investigated if SV or other genetic factors might be linked to methylation differences across ASE secretome genes. We compared TE occupancy in 5' and 3' flanking regions of ASE genes with the expectation that fungal TEs are typically heavily methylated (Bewick et al. 2019), but no significant difference was found between higher- and lower-expressed alleles (Mann-Whitney U tests; Supplemental Fig. S14). Permutation tests also revealed depletions of ASE events within and nearby whole-genome SVs (Fig. 5C). No correlation between allele sequence divergence and expression differences of ASE secretome genes was found, except a weak positive correlation detected at 8 dpi (Spearman's Rank-Order correlation tests; Supplemental Fig. S15).
