# Supplementary material for: Long-read genomics reveal extensive nuclear-specific evolution and allele-specific expression in a dikaryotic fungus
Source: Genome Res. 2025 Jun;35(6):1364–76. doi: 10.1101/gr.280359.124 (PMC12129025; doi:10.1101/gr.280359.124)
Supplement: Supplement 5 [file Supplemental_Table_S1.pdf]

Supplemental Table S1. Summary statistics of the raw Verkko assembly of *Pst*104E.

**Assembly statistics**	**Raw Verkko assembly**
Assembly size (bp)	168,220,354 bp
# contigs	355
# scaffolds	1
# haplotype-assigned contigs/scaffolds	40 (20 per haplotype)
GC content	44.12%
N/L50	4.5Mbp/17
N/L90	125Kbp/39
T2T contigs	26/36
T2T scaffolds	1/36
Complete BUSCOs*	92.5%
Fragmented BUSCOs	1.0%
Missing BUSCOs	6.5%
Merqury QV	49.0

*basidiomycota_odb10 lineage dataset from BUSCO v5.5.0. # BUSCOs = 1764.
