# Supplementary material for: Long-read genomics reveal extensive nuclear-specific evolution and allele-specific expression in a dikaryotic fungus
Source: Genome Res. 2025 Jun;35(6):1364–76. doi: 10.1101/gr.280359.124 (PMC12129025; doi:10.1101/gr.280359.124)
Supplement: Supplement 6 [file Supplemental_Table_S2.pdf]

Supplemental Table S2. Summary statistics of the time-course ONT direct cDNA sequencing dataset for *Pst*104E. This includes number of reads, read length, percentage of read quality above Q15, and percentage of reads mapped to the *Pst*104E assembly, etc.

sample_name	condition	rep	#reads	N50	total_bases	q10	q15	%reads mapped to *Pst*104E
Pst104E_U_4dpi_rep1	4 dpi	1	9091024	1312	9919765407	99.6%	74.1%	0.69%
Pst104E_U_4dpi_rep2	4 dpi	2	10778794	1270	11332646727	99.6%	74.5%	0.94%
Pst104E_U_4dpi_rep3	4 dpi	3	10297394	1310	11190944972	99.6%	74.5%	1.29%
Pst104E_U_4dpi_rep4	4 dpi	4	11018662	1200	10743402830	99.5%	74.5%	0.59%
Pst104E_U_6dpi_rep1	6 dpi	1	4493674	1227	4524045251	99.5%	73.3%	2.66%
Pst104E_U_6dpi_rep2	6 dpi	2	8915962	1383	10023512028	99.5%	74.0%	7.12%
Pst104E_U_6dpi_rep3	6 dpi	3	9059496	1296	9582138550	99.5%	74.2%	4.39%
Pst104E_U_6dpi_rep4	6 dpi	4	9100347	1244	9324707083	99.5%	73.5%	9.97%
Pst104E_U_8dpi_rep1	8 dpi	1	8445183	1347	9319492816	99.5%	75.5%	19.70%
Pst104E_U_8dpi_rep2	8 dpi	2	9714725	1310	10345871707	99.4%	73.5%	16.77%
Pst104E_U_8dpi_rep3	8 dpi	3	8782186	1318	9409739196	99.5%	75.2%	20.62%
Pst104E_U_8dpi_rep4	8 dpi	4	8314628	1285	8897869617	99.5%	72.9%	11.66%
Pst104E_U_10dpi_rep1	10 dpi	1	4212699	1372	4770645732	99.5%	72.7%	64.36%
Pst104E_U_10dpi_rep2	10 dpi	2	2857068	1288	2987213599	99.3%	71.7%	63.55%
Pst104E_U_10dpi_rep3	10 dpi	3	4548464	1298	4761380316	99.4%	72.1%	60.55%
Pst104E_U_10dpi_rep4	10 dpi	4	4523998	1294	4752937074	99.4%	72.9%	69.10%
Pst104E_U_12dpi_rep1	12 dpi	1	5464381	1078	4771877423	99.2%	73.3%	59.24%
Pst104E_U_12dpi_rep2	12 dpi	2	5060939	1273	5231504385	99.4%	73.1%	38.73%
Pst104E_U_12dpi_rep3	12 dpi	3	4807731	1171	4514628923	99.3%	71.8%	41.43%
Pst104E_U_12dpi_rep4	12 dpi	4	8365833	952	6238974350	99.1%	69.9%	58.18%
Pst104E_U_UG_rep1	UG	1	4586868	1266	4709409947	99.4%	70.6%	94.44%
Pst104E_U_UG_rep2	UG	2	4418105	1281	4720936470	99.4%	71.1%	94.63%
Pst104E_U_UG_rep3	UG	3	3894580	1329	4327384423	99.5%	71.3%	94.78%
Pst104E_U_UG_rep4	UG	4	4479789	1289	4780110383	99.4%	71.6%	93.47%
