# Supplementary material for: Long-read genomics reveal extensive nuclear-specific evolution and allele-specific expression in a dikaryotic fungus
Source: Genome Res. 2025 Jun;35(6):1364–76. doi: 10.1101/gr.280359.124 (PMC12129025; doi:10.1101/gr.280359.124)
Supplement: Supplement 7 [file Supplemental_Table_S3.pdf]

Supplemental Table S3. Metadata and citations for all public Illumina RNA-seq datasets for three different *Pst* isolates (*Pst*104E, *Pst*87/66 and CYR32) used for transcriptome assembly and gene annotations in this study.

Pst **strain**	**host plant**	**host plant cultivar**	**spore type**	**condition**	**rep**	**BioProject**	**citation**
Pst 87/66	wheat	Vuka	Urediniospores	1dpi	1	PRJEB12497	Dobon et al. 2016
Pst 87/66	wheat	Vuka	Urediniospores	1dpi	2	PRJEB12497	Dobon et al. 2016
Pst 87/66	wheat	Vuka	Urediniospores	1dpi	3	PRJEB12497	Dobon et al. 2016
Pst 87/66	wheat	Vuka	Urediniospores	2dpi	1	PRJEB12497	Dobon et al. 2016
Pst 87/66	wheat	Vuka	Urediniospores	2dpi	2	PRJEB12497	Dobon et al. 2016
Pst 87/66	wheat	Vuka	Urediniospores	2dpi	3	PRJEB12497	Dobon et al. 2016
Pst 87/66	wheat	Vuka	Urediniospores	3dpi	1	PRJEB12497	Dobon et al. 2016
Pst 87/66	wheat	Vuka	Urediniospores	3dpi	2	PRJEB12497	Dobon et al. 2016
Pst 87/66	wheat	Vuka	Urediniospores	3dpi	3	PRJEB12497	Dobon et al. 2016
Pst 87/66	wheat	Vuka	Urediniospores	5dpi	1	PRJEB12497	Dobon et al. 2016
Pst 87/66	wheat	Vuka	Urediniospores	5dpi	2	PRJEB12497	Dobon et al. 2016
Pst 87/66	wheat	Vuka	Urediniospores	5dpi	3	PRJEB12497	Dobon et al. 2016
Pst 87/66	wheat	Vuka	Urediniospores	7dpi	1	PRJEB12497	Dobon et al. 2016
Pst 87/66	wheat	Vuka	Urediniospores	7dpi	2	PRJEB12497	Dobon et al. 2016
Pst 87/66	wheat	Vuka	Urediniospores	7dpi	3	PRJEB12497	Dobon et al. 2016
Pst 87/66	wheat	Vuka	Urediniospores	9dpi	1	PRJEB12497	Dobon et al. 2016
Pst 87/66	wheat	Vuka	Urediniospores	9dpi	2	PRJEB12497	Dobon et al. 2016
Pst 87/66	wheat	Vuka	Urediniospores	9dpi	3	PRJEB12497	Dobon et al. 2016
Pst 87/66	wheat	Vuka	Urediniospores	11dpi	1	PRJEB12497	Dobon et al. 2016
Pst 87/66	wheat	Vuka	Urediniospores	11dpi	2	PRJEB12497	Dobon et al. 2016
Pst 87/66	wheat	Vuka	Urediniospores	11dpi	3	PRJEB12497	Dobon et al. 2016
Pst 87/66	wheat	-	Urediniospores	germinated spores	1	PRJEB12497	Dobon et al. 2016
Pst 87/66	wheat	-	Urediniospores	germinated spores	2	PRJEB12497	Dobon et al. 2016
Pst 87/66	wheat	-	Urediniospores	germinated spores	3	PRJEB12497	Dobon et al. 2016
Pst104E	wheat	-	Urediniospores	ungerminated spores	1	PRJNA396589	Schwessinger et al. 2018
Pst104E	wheat	-	Urediniospores	ungerminated spores	2	PRJNA396589	Schwessinger et al. 2018
Pst104E	wheat	-	Urediniospores	ungerminated spores	3	PRJNA396589	Schwessinger et al. 2018
Pst104E	wheat	-	Urediniospores	germinated spores	1	PRJNA396589	Schwessinger et al. 2018
Pst104E	wheat	-	Urediniospores	germinated spores	2	PRJNA396589	Schwessinger et al. 2018

Pst104E	wheat	-	Urediniospores	germinated spores	3	PRJNA396589	Schwessinger et al. 2018
Pst104E	wheat	Morocco	Urediniospores	hautoria-enriched fraction	1	PRJNA396589	Schwessinger et al. 2018
Pst104E	wheat	Morocco	Urediniospores	hautoria-enriched fraction	2	PRJNA396589	Schwessinger et al. 2018
Pst104E	wheat	Morocco	Urediniospores	hautoria-enriched fraction	3	PRJNA396589	Schwessinger et al. 2018
Pst104E	wheat	Morocco	Urediniospores	6dpi	1	PRJNA396589	Schwessinger et al. 2018
Pst104E	wheat	Morocco	Urediniospores	6dpi	2	PRJNA396589	Schwessinger et al. 2018
Pst104E	wheat	Morocco	Urediniospores	6dpi	3	PRJNA396589	Schwessinger et al. 2018
Pst104E	wheat	Morocco	Urediniospores	9dpi	1	PRJNA396589	Schwessinger et al. 2018
Pst104E	wheat	Morocco	Urediniospores	9dpi	2	PRJNA396589	Schwessinger et al. 2018
Pst104E	wheat	Morocco	Urediniospores	9dpi	3	PRJNA396589	Schwessinger et al. 2018
CYR32	barberry	Berberis shensiana Ahrendt	Basidiospores	4dpi	3	PRJNA637808	Zhao et al. 2021
CYR32	barberry	Berberis shensiana Ahrendt	Basidiospores	4dpi	2	PRJNA637808	Zhao et al. 2021
CYR32	barberry	Berberis shensiana Ahrendt	Basidiospores	4dpi	1	PRJNA637808	Zhao et al. 2021
CYR32	barberry	Berberis shensiana Ahrendt	Basidiospores	3dpi	3	PRJNA637808	Zhao et al. 2021
CYR32	barberry	Berberis shensiana Ahrendt	Basidiospores	3dpi	2	PRJNA637808	Zhao et al. 2021
CYR32	barberry	Berberis shensiana Ahrendt	Basidiospores	3dpi	1	PRJNA637808	Zhao et al. 2021
CYR32	wheat	Mingxian 169	Urediniospores	2dpi	3	PRJNA637808	Zhao et al. 2021
CYR32	wheat	Mingxian 169	Urediniospores	2dpi	2	PRJNA637808	Zhao et al. 2021
CYR32	wheat	Mingxian 169	Urediniospores	2dpi	1	PRJNA637808	Zhao et al. 2021
CYR32	wheat	Mingxian 169	Urediniospores	1dpi	3	PRJNA637808	Zhao et al. 2021
CYR32	wheat	Mingxian 169	Urediniospores	1dpi	2	PRJNA637808	Zhao et al. 2021
CYR32	wheat	Mingxian 169	Urediniospores	1dpi	1	PRJNA637808	Zhao et al. 2021
