# Supplementary material for: Long-read genomics reveal extensive nuclear-specific evolution and allele-specific expression in a dikaryotic fungus
Source: Genome Res. 2025 Jun;35(6):1364–76. doi: 10.1101/gr.280359.124 (PMC12129025; doi:10.1101/gr.280359.124)
Supplement: Supplement 8 [file Supplemental_Table_S4.pdf]

**Supplemental Table S4.** Information on the top 25 of 97 hemizygous *Avr* candidates identified via DESeq2 differential gene expression analysis based on ONT cDNA transcript abundance quantified with bambu. Columns are listed in the following order: gene identifier, expression $\log_2$ fold change in all *in planta* conditions (4 to 12 days post infection) relative to the dormant ungerminated urediniospores (UG) condition, full CDS lengths, and the associated functional annotations.

Full-length table is available on Zenodo (see Data Access in the manuscript).

| **gene_identifier** | **4dpi_lfc** | **6dpi_lfc** | **8dpi_lfc** | **10dpi_lfc** | **12dpi_lfc** | **cds_length** | **functional_annotation** |
|---|---|---|---|---|---|---|---|
| Pst104E137_005603 | 7.848 | 11.679 | 10.614 | 8.794 | 7.764 | 456 | SECRETED:SignalP(1-25); |
| Pst104E137_014999 | 8.855 | 10.227 | 10.280 | 9.884 | 9.160 | 252 | SECRETED:SignalP(1-22),ingenannot:predicted_effector; |
| Pst104E137_019128 | NULL | 9.278 | 8.971 | 8.542 | 8.102 | 231 | ingenannot:predicted_effector,SECRETED:SignalP(1-24); |
| Pst104E137_021272 | 8.288 | 10.188 | 10.093 | 8.509 | 8.147 | 1074 | SECRETED:SignalP(1-25); |
| Pst104E137_029659 | NULL | 9.089 | 8.149 | 9.752 | 9.241 | 297 | ingenannot:predicted_effector,SECRETED:SignalP(1-19); |
| Pst104E137_021690 | 8.113 | 9.954 | 10.598 | 10.319 | 9.442 | 1659 | SECRETED:SignalP(1-24); |
| Pst104E137_021528 | 7.395 | 10.355 | 10.136 | 8.314 | 7.723 | 1041 | SECRETED:SignalP(1-29); |
| Pst104E137_014762 | 8.924 | 8.180 | 8.684 | 7.802 | 7.717 | 330 | SECRETED:SignalP(1-22),ingenannot:predicted_effector; |
| Pst104E137_024713 | 8.078 | 8.841 | 10.131 | 8.634 | 8.290 | 771 | SECRETED:SignalP(1-19); |
| Pst104E137_005001 | 7.785 | 8.728 | 8.687 | 10.130 | 10.490 | 351 | SECRETED:SignalP(1-27); |
| Pst104E137_021794 | 7.716 | 7.765 | 7.931 | 7.026 | 6.068 | 249 | ingenannot:predicted_effector,SECRETED:SignalP(1-19); |
| Pst104E137_020506 | 7.450 | 8.026 | 8.548 | 7.605 | 7.873 | 249 | SECRETED:SignalP(1-20); |
| Pst104E137_022562 | NULL | 7.579 | 7.065 | 5.755 | 5.506 | 219 | SECRETED:SignalP(1-24); |
| Pst104E137_021402 | 8.782 | 6.283 | 9.273 | 7.916 | 9.344 | 585 | SECRETED:SignalP(1-29); |
| Pst104E137_028795 | 6.317 | 8.722 | 8.386 | 6.644 | 6.023 | 624 | SECRETED:SignalP(1-25); |
| Pst104E137_028801 | 6.273 | 8.626 | 8.401 | 6.522 | 6.010 | 624 | SECRETED:SignalP(1-25); |
| Pst104E137_013184 | NULL | 7.446 | 7.539 | 6.960 | 6.618 | 1236 | EggNog:ENOG503NU3V,SECRETED:SignalP(1-31),COG:G;Trehalose-phosphatase; |
| Pst104E137_010232 | 6.139 | 8.717 | 9.859 | 8.903 | 7.545 | 600 | EggNog:ENOG503NUD5,COG:H,SECRETED:SignalP(1-19),ingenannot:predicted_effector; |
| Pst104E137_030210 | NULL | 7.322 | 7.590 | 7.849 | 7.588 | 486 | SECRETED:SignalP(1-22); |
| Pst104E137_029664 | NULL | 7.279 | 7.412 | 6.320 | 5.985 | 348 | SECRETED:SignalP(1-20); |
| Pst104E137_021280 | 6.257 | 8.108 | 8.453 | 7.043 | 6.332 | 1074 | SECRETED:SignalP(1-25); |
| Pst104E137_024997 | 6.139 | 8.196 | 8.413 | 7.857 | 7.273 | 345 | ingenannot:predicted_effector,SECRETED:SignalP(1-24); |
| Pst104E137_019245 | NULL | 7.159 | 7.080 | 6.858 | 6.596 | 183 | SECRETED:SignalP(1-20); |
| Pst104E137_008987 | NULL | 7.138 | 7.372 | 6.153 | 5.207 | 249 | SECRETED:SignalP(1-18),ingenannot:predicted_effector; |
| Pst104E137_018474 | 5.800 | 8.345 | 8.525 | 8.308 | 8.600 | 201 | SECRETED:SignalP(1-18); |
