# Supplementary material for: Long-read genomics reveal extensive nuclear-specific evolution and allele-specific expression in a dikaryotic fungus
Source: Genome Res. 2025 Jun;35(6):1364–76. doi: 10.1101/gr.280359.124 (PMC12129025; doi:10.1101/gr.280359.124)
Supplement: Supplement 9 [file Supplemental_Table_S5.pdf]

Supplemental Table S5. Summary statistics of transposable elements (TEs) identified and classified for each of the nuclear haplotype of the *Pst*104E genome assembly using the REPET pipeline.

TE classification			Haplotype A				Haplotype B			
**class**	**order**	**superfamily**	**full length copies**	**copies**	**coverage (bp)**	**% genome covered**	**full length copies**	**copies**	**coverage (bp)**	**% genome covered**
**Class I**	**Class I total**		**1462**	**14128**	**11734815**	**15.215**	**1579**	**14322**	**11120666**	**14.788**
	LTR	Gypsy	989	10079	8902187	11.542	1106	10050	8169640	10.864
		Copia	275	2164	2019141	2.618	296	2294	2117522	2.816
		?	35	347	72012	0.093	42	403	81239	0.108
	TRIM		96	861	224731	0.291	80	604	108805	0.145
	DIRS		20	234	221390	0.287	14	238	179764	0.239
	LINE		20	233	213926	0.277	13	118	143144	0.190
	LARD		13	166	67002	0.087	22	465	199930	0.266
	SINE		12	29	7396	0.010	3	69	68266	0.091
	Class I Unclassified		2	15	7030	0.009	3	81	52356	0.070
**Class II**	**Class II total**		**2954**	**32237**	**14829353**	**19.227**	**2857**	**30876**	**13621037**	**18.113**
	TIR	hAT	369	3475	2310708	2.996	331	3000	2019611	2.686
		MuDR	133	1967	1436788	1.863	118	2440	1489670	1.981
		PIF	194	1784	1102250	1.429	153	1766	1058009	1.407
		Tc1	186	2159	846786	1.098	224	2205	897857	1.194
		CACTA	6	79	261407	0.339	7	61	236785	0.315
		Tc1-Mariner	3	392	159928	0.207	1	7	4341	0.006
		P	31	359	122592	0.159	25	229	63974	0.085
		PIF-Harbinger	3	269	96234	0.125	0	0	0	0.000
		?	640	7266	3238925	4.199	628	7727	3278137	4.359
	MITE		685	3539	1122619	1.456	755	4187	1295540	1.723
	Helitron		62	1163	692793	0.898	41	836	610412	0.812
	?	Academ	6	308	158471	0.205	11	255	195962	0.261
	Maverick		0	0	0	0.000	2	2	2176	0.003
	Class II Unclassified		636	9477	3279852	4.252	561	8161	2468563	3.283
**Unclassified**			2656	17527	8266876	10.718	2859	18181	8252730	10.974
**Total**			7072	63892	34831044	**45.160**	7295	63379	32994433	**43.876**
