# Supplementary material for: Long-read genomics reveal extensive nuclear-specific evolution and allele-specific expression in a dikaryotic fungus
Source: Genome Res. 2025 Jun;35(6):1364–76. doi: 10.1101/gr.280359.124 (PMC12129025; doi:10.1101/gr.280359.124)
Supplement: Supplement 10 [file Supplemental_Table_S6.pdf]

Supplemental Table S6. Coordinates and length of the inferred centromeres and the centromere dip region (CDR). The CDR midpoint was used to calculate its relative position by dividing its coordinate by the centromere length, then compared to the relative CDR position on its homologous centromere.

centromere	centromere_start	centromere_end	centromere_length_kbp	CDR_start	CDR_end	CDR_midpoint	CDR_length_kbp
Cen1A	1718007	2036434	318.4	1880671	1900126	1890398	19455
Cen1B	1679640	2024448	344.8	1865584	1893197	1879390	27613
Cen2A	2244206	2552847	308.6	2375247	2400415	2387831	25168
Cen2B	2222042	2477982	255.9	2321099	2342188	2331643	21089
Cen3A	1593990	1877227	283.2	1744378	1766144	1755261	21766
Cen3B	1462360	1757066	294.7	1495879	1514490	1505184	18611
Cen4A	1373984	1661688	287.7	1523193	1548108	1535650	24915
Cen4B	1360510	1637020	276.5	1455825	1482910	1469367	27085
Cen5A	1963324	2399107	435.8	2100887	2122363	2111625	21476
Cen5B	1989903	2199877	210.0	2051583	2080156	2065869	28573
Cen6A	2325075	2627632	302.6	2428818	2456404	2442611	27586
Cen6B	2253114	2569015	315.9	2347095	2374174	2360634	27079
Cen7A	1434064	1713521	279.5	1567859	1596190	1582024	28331
Cen7B	1458954	1700142	241.2	1577358	1600463	1588910	23105
Cen8A	1055477	1294381	238.9	1160178	1183820	1171999	23642
Cen8B	1167966	1377956	210.0	1267312	1290927	1279119	23615
Cen9A	1150502	1367818	217.3	1219789	1247441	1233615	27652
Cen9B	1231732	1482851	251.1	1338503	1373589	1356046	35086
Cen10A	774637	1010882	236.2	877259	910292	893775	33033
Cen10B	744571	1100491	355.9	935821	959425	947623	23604
Cen11A	657123	1194917	537.8	912239	943166	927702	30927
Cen11B	639298	1173308	534.0	879916	909011	894463	29095
Cen12A	1074207	1319319	245.1	1203983	1229055	1216519	25072
Cen12B	1347209	1612234	265.0	1486409	1512754	1499581	26345
Cen13A	867062	1192301	325.2	1143572	1156857	1150214	13285
Cen13B	807092	1138885	331.8	982961	1015975	999468	33014
Cen14A	1156087	1498910	342.8	1169104	1199437	1184270	30333
Cen14B	1094011	1431560	337.5	1104573	1134640	1119606	30067
Cen15A	532364	899633	367.3	733485	757343	745414	23858
Cen15B	514404	781823	267.4	661954	680253	671103	18299

Cen16A	708039	1002809	294.8	854345	878222	866283	23877
Cen16B	684647	964992	280.3	814026	843666	828846	29640
Cen17A	520320	878821	358.5	745114	765376	755245	20262
Cen17B	459377	751483	292.1	632227	647714	639970	15487
Cen18A	1158321	1413905	255.6	1238505	1256803	1247654	18298
Cen18B	1188275	1437271	249.0	1269871	1286134	1278002	16263
