# Supplementary material for: Long-read genomics reveal extensive nuclear-specific evolution and allele-specific expression in a dikaryotic fungus
Source: Genome Res. 2025 Jun;35(6):1364–76. doi: 10.1101/gr.280359.124 (PMC12129025; doi:10.1101/gr.280359.124)
Supplement: Supplement 11 [file Supplemental_Table_S7.pdf]

Supplemental Table S7. Permutation test results for centromeric TE superfamily enrichment or depletion, conducted for each chromosome (Only Chromosome 1A shown as example). The test statistic is the TE coverage difference between centromeric and non-centromeric region. TE locations are randomly shuffled along each chromosome in 5,000 permutations to generate a null distribution, enabling a two-tailed test for the statistical significance of centromeric enrichment or depletion per TE superfamily. P-values, defined as the proportion of permuted results equal to or more extreme than the observed, were adjusted for multiple testing using <5% FDR.

Full-length table is available on Zenodo (see Data Access in the manuscript).

chr	TE_Class:Order:Superfamily	Observed_cov_diff (centromere-noncentromere)	perm_test_p-value	perm_test_p-value_FDRcorr
chr1A	noCat	4.898384437	0.0726	0.4094
chr1A	ClassII:TIR:?	7.763363833	0.0032	0.0533
chr1A	ClassI:LTR:Copia	4.295907268	0.0272	0.2242
chr1A	ClassII:TIR:Tc1	-1.355232296	0.1074	0.4797
chr1A	ClassII:TIR:MuDR	6.494198954	0.0126	0.1246
chr1A	ClassII:TIR:PIF	-1.691663652	0.1928	0.6623
chr1A	ClassII:TIR:hAT	1.590364353	0.5442	1.0000
chr1A	ClassI:LTR:Gypsy	13.64832501	0.0092	0.0986
chr1A	ClassI:TRIM:?	-0.316991877	0.1788	0.6307
chr1A	ClassII:Helitron:?	-0.390449522	0.1378	0.5439
chr1A	ClassII:?:?	2.72896062	0.0682	0.4038
chr1A	ClassII:TIR:P	-0.454162013	0.2954	0.8411
chr1A	ClassI:LINE:?	-0.037031585	0.8246	1.0000
chr1A	ClassII:?:Academ	-0.156263068	0.451	0.9861
chr1A	ClassII:TIR:Tc1-Mariner	-0.19248098	0.405	0.9619
chr1A	ClassII:TIR:PIF-Harbinger	0.027408346	0.6694	1.0000
chr1A	ClassII:MITE:?	1.069760163	0.0982	0.4676
chr1A	ClassI:LTR:?	0.090903142	0.367	0.9357
chr1A	ClassI:SINE:?	-0.008893636	0.9998	1.0000
chr1A	ClassI:DIRS:DIRS	1.116071653	0.0342	0.2544
chr1A	ClassII:Helitron:Helitron	2.494107023	0.0746	0.4094
chr1A	ClassI:LARD:?	-0.080230594	0.8444	1.0000
chr1A	ClassI:?:?	0	1	1.0000
chr1A	ClassII:TIR:CACTA	-0.061063323	0.952	1.0000
chr1A	ClassII:Maverick:?	0	1	1.0000
