# Supplementary material for: Long-read genomics reveal extensive nuclear-specific evolution and allele-specific expression in a dikaryotic fungus
Source: Genome Res. 2025 Jun;35(6):1364–76. doi: 10.1101/gr.280359.124 (PMC12129025; doi:10.1101/gr.280359.124)
Supplement: Supplement 12 [file Supplemental_Table_S8.pdf]

Supplemental Table S8. Information on the rarer rDNA subtype variants (#1.1-1.9 and #2.1-2.3) identified via calling low-frequency SNPs from ONT duplex alignment against the two dominant rDNA subtypes #1 and #2. The SNP positions and the overlapping rRNA gene annotations are listed in the top two rows. Point mutations were scored as “reference>alternate(SNP depth)”. SNP combinations for each subtype variant was visually determined in the IGV alignment. SNP depth was used to estimate their copy number by normalising it against the mode value of genome-wide per-base coverage depth.

	**Annotation**	**18S**				**25S**	**5S**	**IGS2**			**Estimated copy number**
	**SNP position**	**320**	**536**	**740**	**1079**	**2975**	**6923**	**7049**	**8542**	**8744**	
**rDNA subtype #1**	**1**										140
	**1.1**	C>T(45)									1
	**1.2**	C>T(45)				G>A(53)					1
	**1.3**		G>A(1203)								26
	**1.4**		G>A(1203)		C>T(195)						6
	**1.5**		G>A(1203)					G>A(285)			4
	**1.6**			G>A(749)							23
	**1.7**						G>A(60)				2
	**1.8**							G>A(285)			4
	**1.9**								T>C(59)	G>C(58)	2

	**Annotation**	**18S**			**Estimated copy number**
	**SNP position**	**636**	**1196**	**1395**	
**rDNA subtype #2**	**2**				195
	**2.1**	C>A(498)			15
	**2.2**		C>T(31)		1
	**2.3**			C>T(429)	13
