# Supplementary material for: Long-read genomics reveal extensive nuclear-specific evolution and allele-specific expression in a dikaryotic fungus
Source: Genome Res. 2025 Jun;35(6):1364–76. doi: 10.1101/gr.280359.124 (PMC12129025; doi:10.1101/gr.280359.124)
Supplement: Supplement 13 [file Supplemental_Table_S9.pdf]

Supplemental Table S9. SyRI summary statistics of the structural variations (SVs), syntenic and unaligned (non-syntenic) regions identified between haplotypes.

		Event size within each alignment block (bp)				Haplotype A		Haplotype B	
**Event type**	**Event count**	Mean	Min	Median	Max	Total bp covered	%covered	Total bp covered	%covered
Inversion	19	15203.8	1285.0	4698.5	14350.3	299021	0.39%	278724	0.37%
Translocation	408	7980.1	530.0	3654.5	5989.8	3329389	4.32%	3182372	4.23%
Duplication	1734	3871.5	503.0	2623.5	5383.0	4223219	5.48%	2490040	3.31%
Indel (>=50bp)	1024	1139.9	50.0	176.5	475.0	530713	0.69%	636495	0.85%
Syntenic region	1317	45641.9	547.5	29169.0	65204.5	60169458	78.01%	60051366	79.86%
Not aligned	2703	6959.6	1.0	3222.0	8737.0	9726962	12.61%	9084758	12.08%
