# Supplementary material for: Long-read genomics reveal extensive nuclear-specific evolution and allele-specific expression in a dikaryotic fungus
Source: Genome Res. 2025 Jun;35(6):1364–76. doi: 10.1101/gr.280359.124 (PMC12129025; doi:10.1101/gr.280359.124)
Supplement: Supplement 14 [file Supplemental_Table_S10.pdf]

**Supplemental Table S10.** Permutation test results for the enrichment or depletion of different genomic features (TEs, genes, secretome/effector genes and allele-specific expressed genes) within different SV types conducted for whole genome. Statistical significance was assessed through two-tailed permutation tests, as described above for centromeric TE analysis.

| Genomic feature | SV category | Observed feature coverage diff within vs outside of given SV category | permutation test p-value | permutation test p-value (FDR corrected) |
|---|---|---|---|---|
| Transposable element | All SVs | 15.50793144 | 0 | 0 |
| | Indel (>=50bp) | 6.912209381 | 0 | 0 |
| | Inversion | 22.38842466 | 0 | 0 |
| | Translocation | 21.66703971 | 0 | 0 |
| | Duplication | 42.50530291 | 0 | 0 |
| Gene | All SVs | -1.235425292 | 0 | 0 |
| | Indel (>=50bp) | -5.105776541 | 0.0004 | 0.000571429 |
| | Inversion | -14.38836517 | 0.0016 | 0.002133333 |
| | Translocation | -14.13300059 | 0 | 0 |
| | Duplication | -27.39173382 | 0 | 0 |
| Secretome/effector genes | All SVs | -4.218475088 | 0 | 0 |
| | Indel (>=50bp) | -0.628310186 | 0.1834 | 0.1834 |
| | Inversion | -3.629882291 | 0.0082 | 0.009647059 |
| | Translocation | -1.91632035 | 0 | 0 |
| | Duplication | -3.969194811 | 0 | 0 |
| Allele-specific expressed genes | All SVs | -30.26566839 | 0 | 0 |
| | Indel (>=50bp) | 0.453896011 | 0.1608 | 0.169263158 |
| | Inversion | -1.997613073 | 0.0336 | 0.037333333 |
| | Translocation | -0.716410851 | 0.0052 | 0.0065 |
| | Duplication | -1.500616614 | 0 | 0 |
