# Supplementary material for: Long-read genomics reveal extensive nuclear-specific evolution and allele-specific expression in a dikaryotic fungus
Source: Genome Res. 2025 Jun;35(6):1364–76. doi: 10.1101/gr.280359.124 (PMC12129025; doi:10.1101/gr.280359.124)
Supplement: Supplement 15 [file Supplemental_Table_S11.pdf]

Supplemental Table S11. Proteinortho results and divergence values for one-to-one allele pairs identified between *Pst*104E haplotypes A and B (15 out of 10,852 one-to-one allele pairs shown as examples). Identified hits from the Proteinortho output table were filtered based on e-values < 0.05 for both ways. Synonymous (dS) and non-synonymous (dN) divergence values along with CDS and protein Levenshtein distances were calculated. A gene pair was determined to be heterozygous biallelic if either its dS or dN value was greater than zero.

Full-length table is available on Zenodo (see Data Access in the manuscript).

Target	Query	evalue_ab	bitscore_ab	evalue_ba	bitscore_ba	protein_levenshtein	cds_levenshtein	dS	dN
Pst104E137_015700	Pst104E137_000068	0	2239	0	2239	0.0007	0.0002	0	0.0003
Pst104E137_015721	Pst104E137_000089	8.78E-79	289	1.12E-78	289	0.229	0.2259	0	0.003
Pst104E137_015723	Pst104E137_000092	3.60E-41	163	3.57E-41	163	0.0125	0.0042	0	0.0062
Pst104E137_015745	Pst104E137_000113	4.27E-67	250	4.24E-67	250	0.0068	0.0023	0	0.0033
Pst104E137_015746	Pst104E137_000114	1.14E-39	158	1.13E-39	158	0.0127	0.0042	0	0.0056
Pst104E137_015751	Pst104E137_000121	4.21E-218	753	4.17E-218	753	0.0026	0.0009	0	0.0012
Pst104E137_015752	Pst104E137_000120	2.21E-200	694	2.20E-200	694	0.0029	0.001	0	0.0014
Pst104E137_015769	Pst104E137_000142	0	1074	0	1074	0.0018	0.0006	0	0.0008
Pst104E137_015781	Pst104E137_000154	0	1712	0	1712	0.0011	0.0004	0	0.0005
Pst104E137_015784	Pst104E137_000156	1.22E-154	542	1.21E-154	542	0.003	0.001	0	0.0014
Pst104E137_015787	Pst104E137_000159	0	2267	0	2267	0.0546	0.0541	0	0.0004
Pst104E137_015805	Pst104E137_000180	3.47E-183	637	3.44E-183	637	0.0027	0.0009	0	0.0012
Pst104E137_015819	Pst104E137_000191	5.61E-40	159	5.57E-40	159	0.0128	0.0043	0	0.0062
Pst104E137_015823	Pst104E137_000196	3.46E-58	220	3.44E-58	220	0.009	0.003	0	0.0043
Pst104E137_015866	Pst104E137_000238	1.99E-106	381	1.97E-106	381	0.0054	0.0018	0	0.0026
