# Supplementary material for: Long-read genomics reveal extensive nuclear-specific evolution and allele-specific expression in a dikaryotic fungus
Source: Genome Res. 2025 Jun;35(6):1364–76. doi: 10.1101/gr.280359.124 (PMC12129025; doi:10.1101/gr.280359.124)
Supplement: Supplement 16 [file Supplemental_Table_S12.pdf]

Supplemental Table S12. DESeq2 result table (25 allele pairs differentially expressed at UG shown as example) for allele-specific expression (ASE) analysis conducted on the heterozygous biallelic gene pairs, with haplotype A alleles set as the reference. The resulting |LFC| and FDR adjusted p-values (padj) were used to categorise the allele pairs into different ASE status, as detailed in the Results.

Full-length tables for all six transcript sampling conditions in separate tabs (UG, 4, 6, 8, 10 and 12 dpi) are available on Zenodo (see Data Access in the manuscript).

HeterozygousAllelePairs	baseMean	log2FoldChange	lfcSE	stat	pvalue	padj
Pst104E137_011008:Pst104E137_026571	79.67586726	-5.171912958	0.166444808	-31.0728404	5.61E-212	3.75E-208
Pst104E137_014436:Pst104E137_029861	70.19465192	-2.963624053	0.105174388	-28.1781915	1.08E-174	3.61E-171
Pst104E137_002654:Pst104E137_018166	254.2866031	5.4894674	0.20531772	26.7364522	1.77E-157	3.95E-154
Pst104E137_012361:Pst104E137_027859	133.7525328	-2.28825931	0.090972265	-25.1533729	1.30E-139	2.17E-136
Pst104E137_013539:Pst104E137_029003	97.64653371	2.644922208	0.119175557	22.19349571	3.97E-109	5.30E-106
Pst104E137_010379:Pst104E137_025952	40.90065639	4.541752426	0.22674635	20.03010163	3.01E-89	3.35E-86
Pst104E137_012439:Pst104E137_027936	60.70872035	-3.877697223	0.193952798	-19.9929945	6.34E-89	6.05E-86
Pst104E137_012532:Pst104E137_028025	24.48756761	2.483319205	0.131742798	18.84975297	2.95E-79	2.46E-76
Pst104E137_002655:Pst104E137_018167	94.27067786	5.49994397	0.296770216	18.53266829	1.13E-76	8.35E-74
Pst104E137_011975:Pst104E137_026767	451.1303014	4.005056445	0.21750296	18.41380206	1.02E-75	6.80E-73
Pst104E137_014635:Pst104E137_030036	38.88503726	3.548238985	0.193362835	18.35016011	3.29E-75	2.00E-72
Pst104E137_009243:Pst104E137_024755	286.1323217	5.920435479	0.324758474	18.23027251	2.97E-74	1.65E-71
Pst104E137_011130:Pst104E137_026695	22.32808545	-4.526077088	0.248791252	-18.1922678	5.94E-74	3.05E-71
Pst104E137_013430:Pst104E137_028894	24.60239227	-4.075650688	0.22543492	-18.0790566	4.66E-73	2.22E-70
Pst104E137_002951:Pst104E137_018444	43.53842178	-2.649232256	0.149480636	-17.7229127	2.79E-70	1.24E-67
Pst104E137_004825:Pst104E137_020380	31.19117791	3.050442994	0.174689477	17.4620879	2.79E-68	1.16E-65
Pst104E137_009323:Pst104E137_024836	60.08803483	2.273173954	0.132650752	17.13653277	7.92E-66	3.11E-63
Pst104E137_001962:Pst104E137_017518	27.49497699	8.375301981	0.502463473	16.66847927	2.22E-62	8.24E-60
Pst104E137_000642:Pst104E137_016262	32.01045709	2.326990836	0.140241356	16.59275771	7.86E-62	2.76E-59
Pst104E137_010476:Pst104E137_026048	111.7594466	-1.661784016	0.101576286	-16.3599604	3.69E-60	1.23E-57
Pst104E137_007507:Pst104E137_023040	60.58900939	-2.576981138	0.163497997	-15.7615456	5.72E-56	1.82E-53
Pst104E137_008685:Pst104E137_024070	244.1530891	-6.316770291	0.401353392	-15.7386742	8.21E-56	2.49E-53
Pst104E137_007483:Pst104E137_023020	38.14765271	2.536778067	0.162911618	15.57149883	1.14E-54	3.30E-52
Pst104E137_005533:Pst104E137_021095	11.26500526	6.540233278	0.432174002	15.13333345	9.76E-52	2.72E-49
Pst104E137_003031:Pst104E137_018527	68.63633321	5.746331247	0.386301785	14.87523865	4.77E-50	1.28E-47
