# Supplementary material for: Long-read genomics reveal extensive nuclear-specific evolution and allele-specific expression in a dikaryotic fungus
Source: Genome Res. 2025 Jun;35(6):1364–76. doi: 10.1101/gr.280359.124 (PMC12129025; doi:10.1101/gr.280359.124)
Supplement: Supplement 17 [file Supplemental_Table_S13.pdf]

Supplemental Table S13. Two-by-two contingency table of counts of ASE versus non-ASE allele pairs that have at least one allele annotated as secretome gene or BUSCO. Fisher's exact test (two-sided) was applied to obtain the odds ratio and Fisher's exact p-value, adjusted for multiple testing using <5% FDR. UG: ungerminated spores; dpi: days post infection.

**UG**		
	ASE pairs	non-ASE pairs
Secretome	107	828
BUSCO	14	1197
Odds ratio=11.05; Fisher's exact p-val=1.39-e25		

**4 dpi**		
	ASE pairs	non-ASE pairs
Secretome	13	388
BUSCO	9	1189
Odds ratio=4.43; Fisher's exact p-val=6.62e-04		

**6 dpi**		
	ASE pairs	non-ASE pairs
Secretome	44	687
BUSCO	12	1198
Odds ratio=6.39; p-val=4.20e-10		

**8 dpi**		
	ASE pairs	non-ASE pairs
Secretome	85	850
BUSCO	12	1199
Odds ratio=9.99; Fisher's exact p-val=6.26e-20		

**10 dpi**		
	ASE pairs	non-ASE pairs
Secretome	104	831
BUSCO	10	1201
Odds ratio=15.03; Fisher's exact p-val=2.30e-27		

**12 dpi**		
	ASE pairs	non-ASE pairs
Secretome	92	843
BUSCO	9	1202
Odds ratio=14.58;Fisher's exact p-val=1.74e-24		
